# Terminal Hydride Complex of High-Spin Mn

**DOI:** 10.1021/jacs.4c03310

**Published:** 2024-06-28

**Authors:** Alex Drena, Addison Fraker, Niklas B. Thompson, Peter E. Doan, Brian M. Hoffman, Alex McSkimming

**Affiliations:** † Department of Chemistry, Tulane University, New Orleans, Louisiana 70118, United States; ‡ Department of Chemistry, Northwestern University, Evanston, Illinois 60208, United States; § Chemical Sciences and Engineering Division, Argonne National Laboratory, Lemont, Illinois 60439, United States

## Abstract

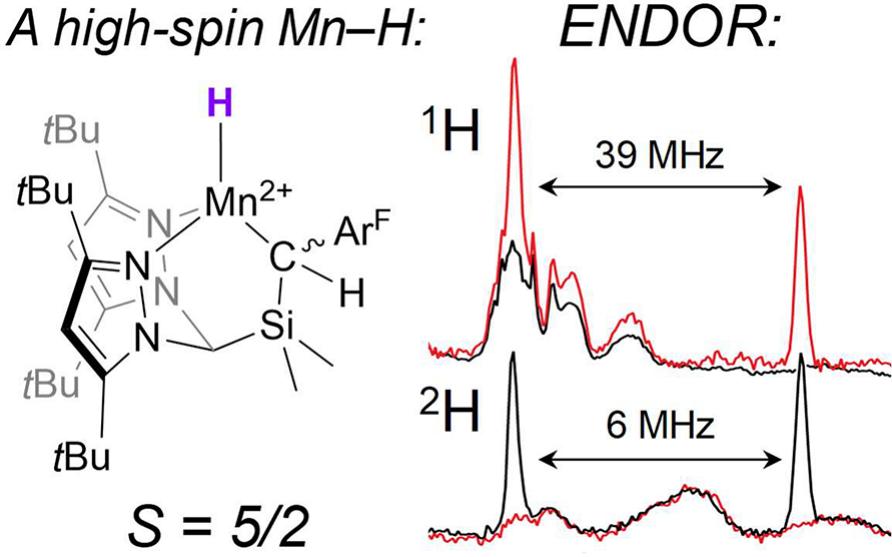

The iron–molybdenum cofactor of nitrogenase (FeMoco) catalyzes fixation of N_2_ via Fe hydride intermediates. Our understanding of these species has relied heavily on the characterization of well-defined 3d metal hydride complexes, which serve as putative spectroscopic models. Although the Fe ions in FeMoco, a weak-field cluster, are expected to adopt locally high-spin Fe^2+/3+^ configurations, synthetically accessible hydride complexes featuring d^5^ or d^6^ electron counts are almost exclusively low-spin. We report herein the isolation of a terminal hydride complex of four-coordinate, high-spin (d^5^; *S* = 5/2) Mn^2+^. Electron paramagnetic resonance and electron–nuclear double resonance studies reveal an unusually large degree of spin density on the hydrido ligand. In light of the isoelectronic relationship between Mn^2+^ and Fe^3+^, our results are expected to inform our understanding of the valence electronic structures of reactive hydride intermediates derived from FeMoco.

## Introduction

**Figure 1:**
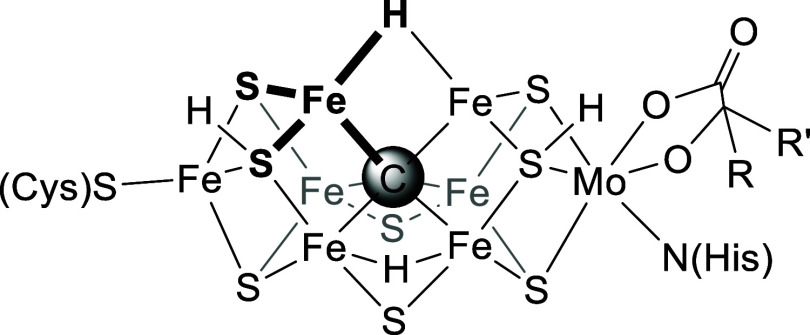
One (of many) possible structures for the E_4_(4H) intermediate of FeMoco.

Transition-metal hydride complexes are ubiquitous throughout synthetic inorganic^1,2^ and bioinorganic^3−7^ chemistry. With respect to the latter, we have maintained a longstanding interest in the Fe–Mo cofactor (FeMoco) of nitrogenase enzymes. This elaborate [Fe_7_S_9_MoC] metallocluster catalyzes the reduction of atmospheric N_2_ to bioavailable NH_3_ as part of the global nitrogen cycle^8^ and, in doing so, supports roughly half the world’s human population.^9^ To bind and activate the highly inert N_2_, FeMoco must first, during turnover, be “primed” via the accumulation of 4H^+^ and 4 e^–^ to afford the so-called E_4_(4H) or “Janus” intermediate (Figure 1).^10^ A paradigm shift in our understanding of FeMoco came with the recognition that E_4_(4H) contains two chemically equivalent Fe hydrides,^11^ whose reductive elimination to H_2_ drives coordination of N_2_ to one or more Fe site(s).^12^

Electron paramagnetic resonance (EPR) and electron–nuclear double resonance (ENDOR) spectroscopies have been central in identifying and characterizing the *S* = 1/2 E_4_(4H) state,^10−14^ as well as other FeMoco intermediates featuring Fe–H bonds.^15^ In this process, structural and electronic assignments have relied heavily on comparisons to synthetic analogues,^16−20^ the majority of which feature strong-field supporting ligands and are, as a result, low-spin.^16,17,19,20^ As useful as these model complexes have been, FeMoco is a relatively weak-field cluster, and, as such, transition-metal hydrides that adopt locally high-spin configurations are more faithful spectroscopic models. Metal hydrides that model the low-coordinate (≤5), weak-field Fe sites within FeMoco are not uncommon.^18,21−44^ Within this group, however, only a handful bear the half-integer spin required for meaningful ENDOR analysis, let alone the d^5^ or d^6^ configurations expected for Fe^2+^ and Fe^3+^, respectively.^18^

FeS clusters, including FeMoco,^45^ have long been known to feature highly delocalized electronic structures.^46^ For example, cuboidal [Fe_4_S_4_]^2+^ clusters, which consist of (formally) two locally high-spin Fe^2+^ and two locally high-spin Fe^3+^ centers, are typically best described as [Fe_4_^2.5+^S_4_]^2+^, i.e., completely valence-delocalized. Recently, however, it has been shown that certain strong-field ligands are able to disrupt electron exchange in such clusters, resulting in valence localization.^47−51^ Most pertinently, a single alkyl ligand causes the bound Fe to adopt partial,^47^ or indeed strong,^48^ Fe^3+^ character. Given the similar donor properties between alkyl and hydrido ligands, it seems possible that Fe–H sites in any FeMoco intermediates might be valence localized, most plausibly as Fe^3+^. We have consequently been drawn to the chemistry of terminal, high-spin Mn^2+^ hydrides, which are isoelectronic to high-spin Fe^3+^ hydrides but considerably less oxidizing and so, presumably, more stable. Although a number of Mn hydrides have been reported, only the polynuclear {[^tBu3^CpMn]_4_[MnH_6_]}^52^ and the recently reported^53^ [(dmpe)_2_MnH(L)]^+^ are open-shell; both are low-spin (*S* = 1/2) at the hydride-bound Mn.

**Scheme 1:**
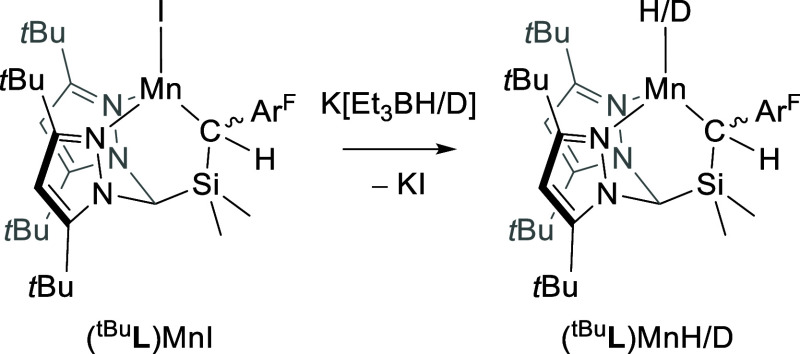
Synthesis of (^tBu^**L**)MnH and Its Deuterium-Labeled Congener Ar^F^ = 3,5-(CF_3_)C_6_H_3_.

Our lab has recently developed a new class of N,N,C heteroscorpionates (^R^**L**; where R denotes the metal-adjacent pyrazolyl substituents, Scheme 1)^54,55^ inspired by the “weak–weak-strong”-field donor environment of the Fe sites in FeMoco (c.f. Figure 1). We postulated that such ligands would be well-suited to support terminal, 3d metal hydrides due to a (i) large, readily modulated steric profile able to hinder dimerization^26−29,32−36,40,43,44^ and (ii) high σ-donicity, which should act to suppress reductive elimination of H_2_ or ^R^**L**H. We report herein the synthesis and characterization of a terminal hydride complex of high-spin (*S* = 5/2) Mn^2+^, (^tBu^**L**)MnH. Q-band EPR measurements show that this complex exists in a novel zero-field splitting regime, while ^1,2^H ENDOR reveals that spin density on the hydride is substantially greater than that for any other previously reported synthetic complex. Our results provide a potentially valuable point of reference for the continued elucidation of the electronic structure of hydride-bound FeMoco states.

## Results

### Synthesis and Characterization

As per our established procedures,^54,55^ deprotonation of ^tBu^**L**H followed by metalation with MnI_2_(THF)_3_ gave the corresponding high-spin Mn^2+^ complex (^tBu^**L**)MnI as a pale yellow, crystalline solid in ∼64% yield (Scheme 1). Addition of 1.5 equiv K[Et_3_BH] to (^tBu^**L**)MnI resulted in an appreciable darkening of the reaction solution, from which the terminal hydride complex (^tBu^**L**)MnH could be isolated (38% yield; see Supporting Information). (^tBu^**L**)MnI and (^tBu^**L**)MnH have very similar ^1^H NMR spectra and solution-state magnetic moments of 6.1 and 6.0 μ_B_, respectively, suggesting a high-spin state at Mn for both with only minimal orbital contributions. We similarly prepared the deuterium-labeled complex (^tBu^**L**)MnD from (^tBu^**L**)MnI and K[Et_3_BD]; the latter reagent was prepared via a new methodology employing cheap and widely available LiD (see Supporting Information), which we anticipate others will find broadly useful in the synthesis of other deuteride complexes.

**Figure 2:**
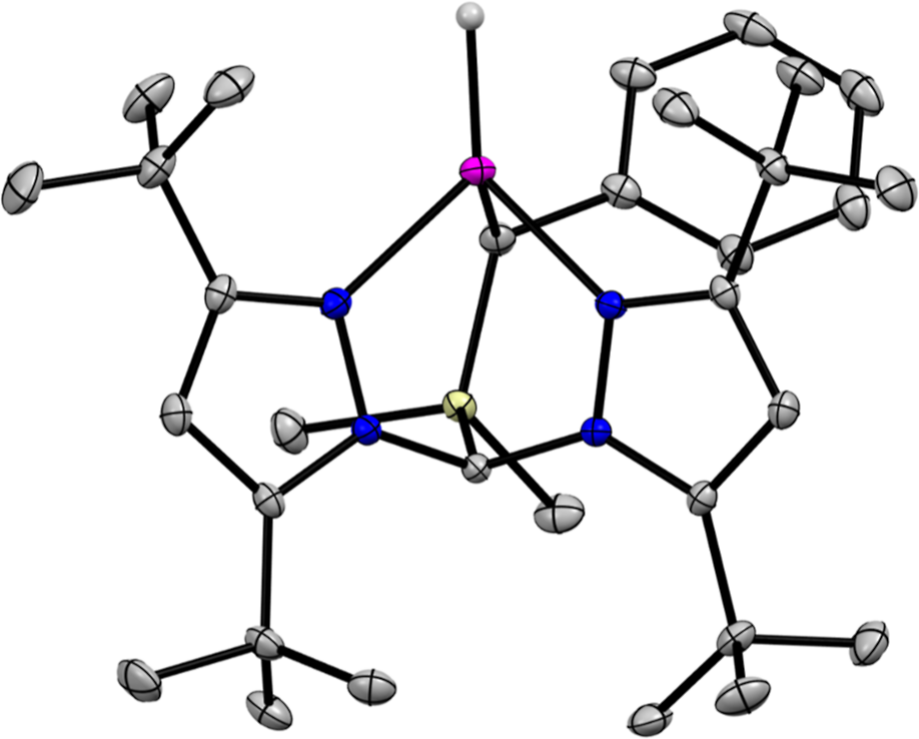
Thermal ellipsoid plot (50%) of (^tBu^**L**)MnH. Pink, blue, yellow, and gray ellipsoids represent Mn, N, Si, and C, respectively. Hydrogen atoms except that bound to Mn, solvent molecules, and CF_3_ groups are omitted for clarity.

The structures of (^tBu^**L**)MnI and (^tBu^**L**)MnH were determined by single-crystal X-ray diffraction (XRD) methods; (^tBu^**L**)MnH is shown in Figure 2. The Mn–^tBu^**L** donor distances are very similar in both complexes and are typical for high-spin Mn^2+^; for example, *d*(Mn–C_alkyl_) = 2.178(2) and 2.215(1) Å for (^tBu^**L**)MnI and (^tBu^**L**)MnH, respectively. The hydrido ligand for (^tBu^**L**)MnH was located in the difference map, and its location was freely refined. The determined position renders the Mn center pseudo-3-fold symmetric about the Mn–H bond, i.e., ∠C_alkyl_–Mn–H ≈ ∠N_pz1_–Mn–H ≈ ∠N_pz2_–Mn–H ≈ 120° with τ_4_ = 0.79. Although care should be taken in interpreting M–H distances without neutron diffraction data, we note that the XRD-determined Mn–H bond length of 1.68(2) Å is similar to that obtained for high-spin, four-coordinate terminal hydride complexes of Co and Fe^18,21,22,25,42^ and is in good agreement with our calculations (see Supporting Information). A weak resonance at 1506 cm^–1^ in the Fourier transform infrared spectrum of (^tBu^**L**)MnH was identified as the Mn–H stretch, which is red-shifted by the expected factor of 1.4 in (^tBu^**L**)MnD. The computed value of 1588 cm^–1^ is somewhat higher but in reasonable agreement. (^tBu^**L**)MnH appears, then, to feature a remarkably, if predictably, weaker Mn–H bond c.f. reported low-spin Mn terminal hydrides [e.g., for [(dmpe)_2_MnH(L)]^+^, ν(Mn–H) > 1700 cm^–1^].^53,56^ A similar observation has been noted, for example, for the high-spin (*S* = 1) TpCoH [ν(Co–H) = 1669 cm^–1^; Tp = tris(pyrazolyl)borate].^21^

### EPR Spectroscopy

**Figure 3:**
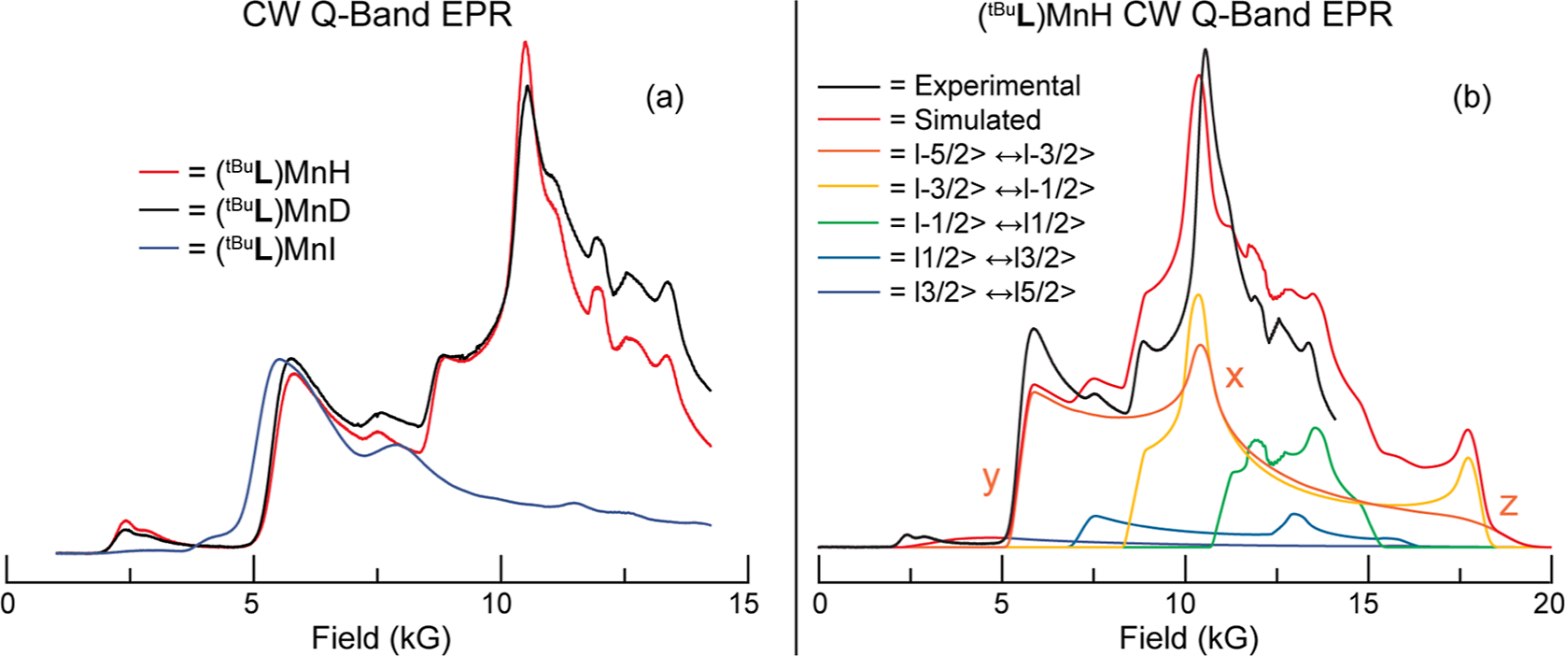
(a) 2 K 35 GHz absorption-display CW EPR spectra of (^tBu^**L**)MnH (red trace), (^tBu^**L**)MnD (black trace), and (^tBu^**L**)MnI (blue trace). Microwave frequency: 34.874 GHz (-H), 34.934 GHz (-D), 34.923 GHz (-I); power attenuation: 20 dB; modulation: 1.6 G; time constant: 64 ms. Spectra have been scaled arbitrarily for clarity. (b) Absorption-display EPR of (^tBu^**L**)Mn(H) (black trace) with simulation (red trace) and the contributions from individual transitions differentiated by color. ZFS principal axes are labeled for the −5/2 → −3/2 manifold. The high-field edge of the experimental spectrum is limited by the available magnetic fields. Parameters used for simulation are *D* = 7600 MHz (0.25 cm^–1^; *E/D* = 0.15; *A* = 250 MHz; *f* = 0.05; *g* = 2.0.

Figure 3a shows the 35 GHz absorption-display EPR spectra of (^tBu^**L**)MnH, (^tBu^**L**)MnD, and (^tBu^**L**)MnI obtained by rapid-passage, CW EPR at 2 K. As expected, the highly articulated spectra of (^tBu^**L**)MnH and (^tBu^**L**)MnD are essentially the same, while that of (^tBu^**L**)MnI differs significantly. Figure 3b compares the experimentally derived 2 K EPR spectrum for (^tBu^**L**)MnH (black trace) and a simulation obtained using EasySpin^57^ (red trace). Despite the presence of substantial structure in the (^tBu^**L**)MnH/D spectra, they could be simulated well with a small range of zero-field splitting (ZFS) parameters. The set of parameters was then optimized by the requirement that both EPR and H/D ENDOR spectra be well-simulated, as described below. The simulations shown in Figure 3b employed ZFS parameters *D* = 7600 MHz (0.25 cm^–1^) and *E/D* = 0.15; in terms of a general ZFS tensor, these parameters are *D* = (3/2)*D*_*z*_ and *E* = 1/2(*D*_*x*_ – *D*_*y*_). In addition, the *g*-tensor is assumed to be isotropic, *g* = 2.0, as the typically spherical spin distribution of the ground S-state of high-spin Mn^2+^ quenches orbital angular momentum, resulting in negligible *g*-anisotropy. Although Mn hyperfine is not resolved, the use of a “standard” isotropic hyperfine value *a*_iso_(^55^Mn) = −250 MHz optimized the simulation. (^tBu^**L**)MnI exhibits a much larger ZFS [*D* = 24,000 MHz (0.83 cm^–1^)] than that of (^tBu^**L**)MnH (Figure S17). The sensitivity of the ZFS in mononuclear Mn^2+^ ions to their ligand environment is well-established,^58−60^ with drastic differences even within the halide series.^61^ In fact, the ZFS parameters for (^tBu^**L**)MnI agree well with those previously reported for other Mn^2+^–I complexes, although it is worth noting that this is the first Mn^2+^ complex with a single iodide ligand so studied.^61−65^

As illustrated in Figure 3b, the spectrum obtained for (^tBu^**L**)MnH is the sum of contributions from the five EPR-allowed transitions (*m*_s_ → *m*_s_ + 1) between electron-spin sublevels (−5/2 ≤ *m*_s_ ≤ 3/2), with intensities of the contributions decreasing with increasing *m*_s_ due to Boltzmann depopulation at 2 K. The breadth and shape of the observed spectra are dominated, respectively, by the axial (*D*) and rhombic (*E*) ZFS parameters. The five EPR-allowed transitions include a roughly isotropic, central −1/2 → +1/2 transition and the four highly anisotropic satellite transitions, which give highly orientation-selective ENDOR responses.^66^ Of particular importance for the ENDOR measurements discussed below, the EPR intensity at the low-field edge of the observed spectrum (∼5 kG ↔ ∼7 kG) is dominated by the contribution from the −5/2 → −3/2 manifold. For *D*, *E* > 0, this edge of this manifold predominantly arises from “single-crystal-like” orientations in which the *Y*-axis of the ZFS tensor is aligned with the external magnetic field,^66,67^ and so a set of related orientations are interrogated in the ∼5–7 kG range. In the higher magnetic field range of ∼11 kG ↔ 15 kG, the EPR spectrum has significant contributions from the *m*_s_ = −5/2 and −3/2 satellite manifolds and the central −1/2 → +1/2 transition. ENDOR spectra collected in both field ranges are reported below.

### Single-Crystal-like ENDOR Spectra along *D*_*Y*_ of the (^tBu^L)MnH/D EPR Envelope

**Figure 4:**
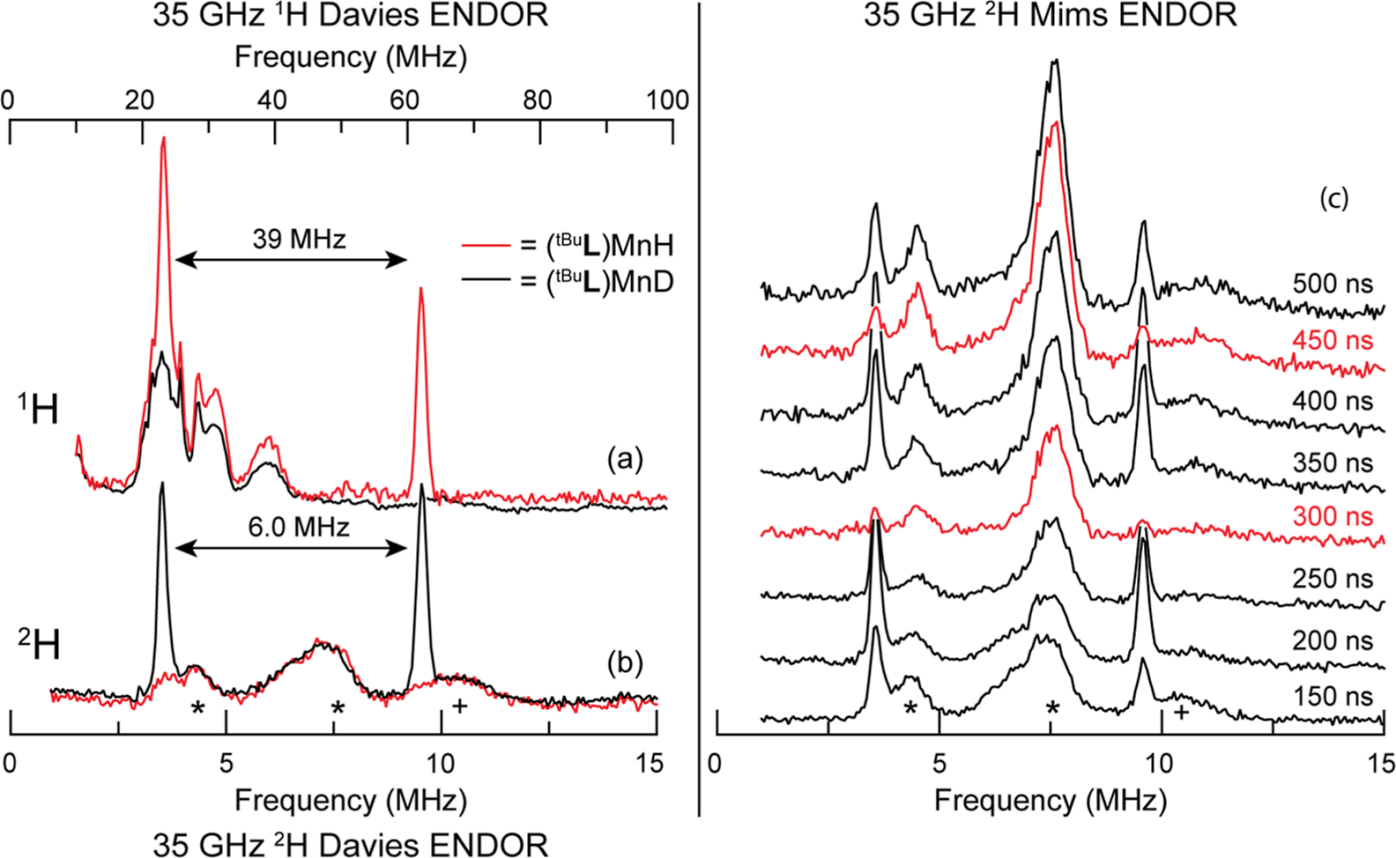
(a) ^1^H and (b) ^2^H Davies ENDOR for (^tBu^**L**)MnH (red trace) and (^tBu^**L**)MnD (black trace) at 5.35 kG and 2 K. The appearance of the frequency axis in (b) is scaled by a factor of 6.5 to account for the difference in *g*_*n*_ between ^1^H and ^2^H. Microwave frequency: 34.6 GHz; microwave pulse length (π): 80 ns (^1^H Davies), 200 ns (^2^H Davies); τ: 600 ns; RF pulse length: 15 μs (^1^H Davies), 60 μs (^2^H Davies); repetition rate: 5 ms. Spectra intensities have been scaled arbitrarily for clarity. (c) 35 GHz ReMims ENDOR of (^tBu^**L**)MnD at 5.35 kG and 2 K. Spectra exhibiting the Mims suppression effect are shown in red. Microwave frequency: 34.6 GHz; microwave pulse length (π/2): 30 ns; τ_1_: varied from 150 to 500 ns as shown in the figure; τ_2_: τ_1_ + 200 ns; RF pulse length: 60 μs; repetition rate: 5 ms. Spectra were scaled to the relative intensity of their observed echo height. 3rd and 5th proton harmonics (*); ^14^N background signal (+).

As thus noted, low-temperature ENDOR at the low-field edge of the EPR envelope selectively probes the −5/2 → −3/2 transition manifold and yields single-crystal-like ENDOR spectra for molecules oriented so that the external field lies along the *Y*-axis of the ZFS *D*-tensor.^66,67^ A ^1^H Davies ENDOR spectrum thus collected, Figure 4a, shows two sharp peaks at 23 and 62 MHz for (^tBu^**L**)MnH (red trace) that are absent in (^tBu^**L**)MnD (black trace); these can be interpreted as corresponding to a ^1^H doublet with an *effective/observed* hyperfine coupling constant of *A′* = −39 MHz, whose magnitude differs from the intrinsic spin-Hamiltonian parameter, as treated below and in greater detail in the Supporting Information. In addition, the procedure for determining the sign of the coupling is presented in the Supporting Information. This effective value for the hydride coupling is confirmed by the ^2^H Davies ENDOR spectrum of (^tBu^**L**)MnD in Figure 4b, which shows a corresponding doublet at 3.5 and 9.5 MHz that is absent in the spectrum for (^tBu^**L**)MnH (red trace), and whose frequency difference yields a matching effective constant of *A*′ = −6.0 MHz, as predicted by the ratio of ^1^H and ^2^H nuclear *g* values [*g*_*n*_(^1^H)/*g*_*n*_(^2^H) = 6.5 = *A*_s_^′^(^1^H)/*A*′(^2^H)]. Note the absence of quadrupole splitting of the narrow ^2^H peaks, as is to be expected for a hydride ion with roughly double-occupancy of its 1s orbital involved in a polar σ bond with Mn.

The assignment of the two ^2^H peaks as a −5/2 → −3/2 hyperfine-split doublet with an effective splitting of *A*′ = −6.0 MHz is verified by the τ dependence of the ReMims (see the Experimental section, Supporting Information) ENDOR response (Figure 4c). ReMims ENDOR follows the same τ-dependence of the signal as Mims ENDOR, with “blind spots” (ENDOR nulls) when *A* (MHz)·τ (μs) = *n*, *n* = 0,1,2,···.^68^ Mims ENDOR is limited by the deadtime of the experiment, while ReMims circumvents this by using a four-pulse-stimulated echo detection subsequence, allowing for the use of much shorter τ values.^69,70^ The resulting suppression of the observed doublet in Figure 4c (red traces) at τ = 300 and 450 ns corresponds to the measured effective hyperfine *A*′ = −6 MHz for *n* = 2 and 3, establishing that these peaks are indeed a doublet separated by this effective hyperfine coupling constant.

The *observed* hyperfine coupling differs from the *intrinsic* coupling because of an “intermediate” magnitude of the ZFS term for (^tBu^**L**)MnH compared to the electron Zeeman at Q-band, neither much smaller nor much larger, which causes significant mixing of *m*_s_ substates. This “intermediate” regime can, of course, be treated by exact calculations such as those performed for simulation with EasySpin, and such simulations are indeed done below. However, the *m*_s_ mixing phenomenon is sufficiently unusual that it is appropriate to illuminate it with a perturbation-theory approach involving first-order modifications to the electron spin *m*_s_ subfunctions by the ZFS interaction. The resultant frequencies for ENDOR transitions involving the two, corrected, lowest-energy electron-spin states when the external field lies along the *Y* direction of the ZFS tensor (low-field edge of the EPR spectrum) can be written in terms of an *m*_s_ formalism (see eqs S1) by incorporating a correction factor, Δ, that accounts for the axial and rhombic contributions of the ZFS term to mixing of the true *m*_s_ substates (eqs 1 and 2). As a result, the observed/effective hyperfine splitting along the *Y*-axis of the ZFS tensor, now specified as A_*Y*_^′^ and defined as the difference in frequencies of the ENDOR doublet, Δν^obs^, is related to the intrinsic hyperfine constant along the *Y*-axis, denoted *A*_*Y*_, and Δ, through eqs 1 and 2.
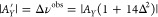
1
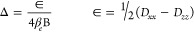
2

This treatment is explained in detail in the Supporting Information. The observed splitting for (^tBu^**L**)MnH, A_*Y*_^′^ = −6.0 MHz, combined with the ZFS parameters determined by the above EPR simulation, gives an intrinsic hyperfine constant *A*_*Y*_ = −5.2 MHz, which is supported by the exact simulations presented in the following section.

### Determination of the Full ^1,2^H Hyperfine Tensor through Analysis of 2D Field-Frequency Patterns of 2H ENDOR Spectra

In contrast to other studies of paramagnetic metal hydrides,^16−20^ the complexity of the EPR spectrum of the *S* = 5/2 (^tBu^**L**)MnH/D complexes (Figure 3) makes it impracticable to collect and analyze a full 2-D ENDOR pattern. However, by collecting spectra over two field ranges, one spanning fields dominated by the lowest-lying −5/2 → −3/2 manifold, now discussed, and a second spanning fields near *g* = 2 (∼12.5 kG at Q-Band), discussed next, and analyzing both patterns through exact simulations (i.e., through full matrix diagonalization) using EasySpin, the full ^1,2^H hyperfine tensor has been determined.

#### ENDOR Spectra at Fields across the Low-Field Edge of the EPR Spectrum

**Figure 5:**
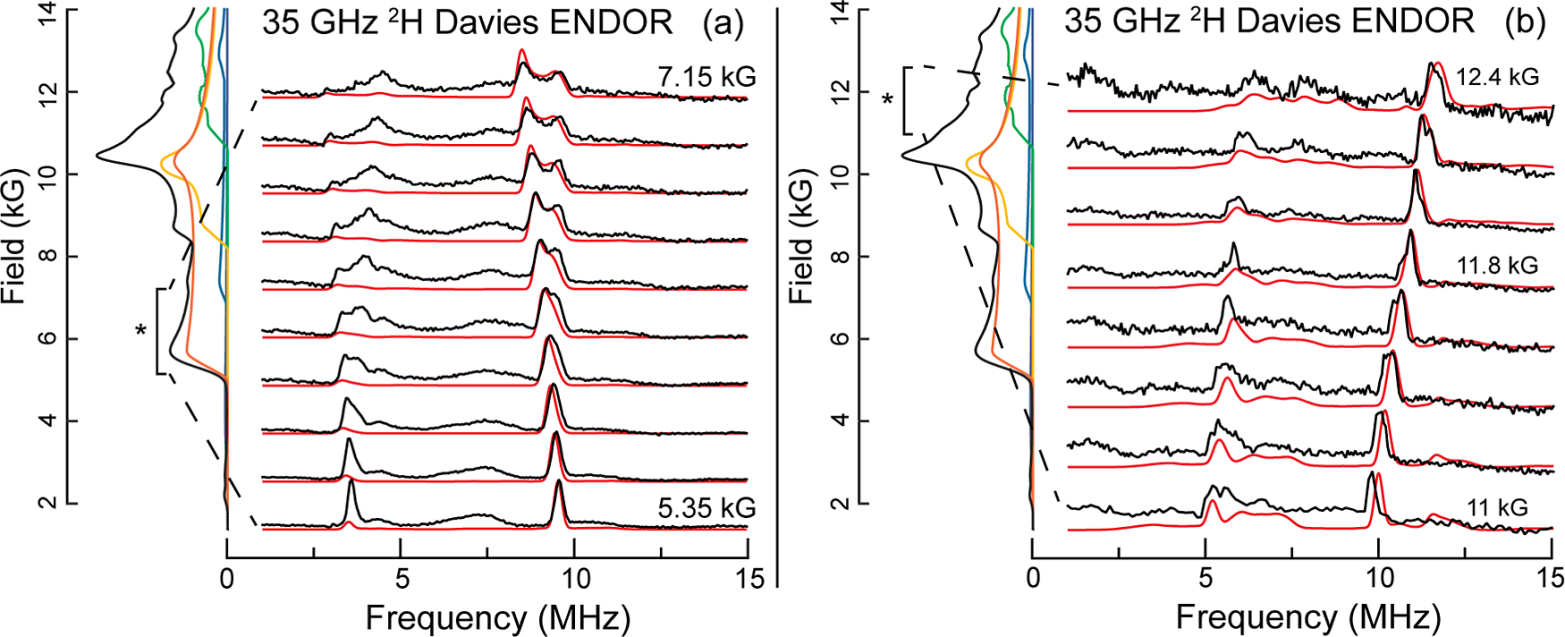
LEFT, i.e., (a) 2 K 35 GHz ^2^H Davies ENDOR 2-D pattern of (^tBu^**L**)MnD. Magnetic field values from 5.35 to 7.15 kG at 0.20 kG intervals. Black: experiment with conditions same as in Figure 4; red: EasySpin simulation with parameters of Figure S15 with ***A*** (^2^H) = [−5.32, −5.32, 1.0] MHz. EPR spectra from Figure 3 are shown on the left with the ENDOR-probed region identified by an asterisk-marked bracket. Spectra normalized for clarity. RIGHT, i.e., (b) 2 K 35 GHz 2-D pattern of ^2^H Davies(^tBu^**L**)MnD ENDOR spectra. Field range: 11.0–12.4 kG, 0.2 kG intervals. Black: experiment with conditions as in Figure 4. Red, EasySpin simulations with the HFI tensor of (a). On left: experimental EPR spectrum from Figure 3, with EasySpin decomposition into *m*_s_ manifolds using parameters of Figure 3b. ENDOR-probed region highlighted by an asterisk-marked bracket. Spectra have been scaled arbitrarily for clarity.

Orientation-selective ENDOR spectra of the 2-D pattern of (^tBu^**L**)MnD, collected from 5.35 to 7.15 kG (Figure 5), show broadening and splitting due to hyperfine anisotropy of the sharp doublet seen at the lowest field. Superimposed on the experimental spectra are EasySpin simulations (in red) calculated with the parameters for the electron-spin Hamiltonian described in Figure 3b and using the axial intrinsic HFI tensor ***A*** (^2^H) = [−5.32, −5.32, 1.0] MHz = *a*_iso_(^2^H) × **1** + ***T*** (^2^H), yielding *a*_iso_(^2^H) = −3.2 MHz [*a*_iso_(^1^H) = −20.9 MHz] and ***T*** (^2^H) = [−2.1, −2.1, 4.2] MHz [***T*** (^1^H) = [−13.7, −13.7, 27.3] MHz].^53^ As anticipated, the simulations show that the unique (*T*_3_) axis lies along the unique (*Z*) axis of the ZFS tensor, which must in turn lie along the Mn–D bond. In addition, we again note that the pattern shows no ^2^H quadrupole splitting, as expected.

Treating ***T*** (^1,2^H) as a dipolar interaction of the H/D nucleus with the spin on Mn gives a rough estimate of Mn–H/D distance: *d*(Mn–H) ≈ 1.8 Å,^71^ in line with structural data and density functional theory (DFT) calculations (see above and below). The isotropic coupling of a hydrogen nucleus to a center with spin *S* is proportional to the 1s-orbital spin density, ρ, through the relationship *a*_iso_ = ρ*a*_0_/2*S*, where *a*_0_ = 1422.7 MHz (^1^H) is the isotropic hyperfine constant for a single (*S* = 1/2) electron in a hydrogen 1s orbital.^72^ This relation and the measured *a*_iso_(^1^H) = −20.9 MHz give ρ = −0.073 spins for the hydride ligand of (^tBu^**L**)MnH, with the negative sign of the spin a result of spin-polarization.

#### ENDOR Spectroscopy at Magnetic Fields in the Vicinity of *g* = 2

Figure S18 shows 35 GHz ^1^H (a) and ^2^H (b) Davies ENDOR spectra collected at 11.8 kG and 2 K for both (^tBu^**L**)MnH and (^tBu^**L**)MnD. Two distinctive ^1^H peaks for (^tBu^**L**)MnH in Figure S18a (red trace) at 37.8 and 70.8 MHz are observed; these form a ^1^H hydride doublet as they match the ^2^H doublet observed for (^tBu^**L**)MnD (Figure S18b, black trace) at frequencies of 5.8 and 10.9 MHz upon accounting for the difference in H/D nuclear *g* values. The Mn–D ^2^H doublet at 11.8 kG is centered at 8.35 MHz and split by an observed hyperfine coupling of *A*′ = −5.1 MHz. In particular, the doublet splittings in Figures 4 and S18 correspond well with the “perpendicular” components of the deuteron hyperfine tensor determined above, *A*(^2^H)_*x,y*_ = −5.32 MHz, as expected for a “powder-like” ENDOR pattern for a hyperfine tensor with |*A*_⊥_| ≫ *A*_||_ ∼ 0. Figure 5b shows the 2-D ENDOR pattern of the ^2^H spectra for (^tBu^**L**)MnD from 11.0 to 12.4 kG. In this range, the contribution of the −1/2 → +1/2 transition to the EPR spectrum is emphasized (Figure 3b), and the ENDOR spectrum shows strong, well-defined signals from this manifold; signals from the other, Boltzmann-depopulated manifolds are very weak and broad because of the poor orientation selection. The “field-evolution” of the −1/2 → +1/2 ^2^H doublet in Figure 5b is well reproduced by EasySpin simulations using the spin-Hamiltonian hyperfine tensor given above. The simulation, which is the sum of ENDOR responses from all orientations and transitions that contribute to the EPR spectrum at the given magnetic field, corroborates that the observed well-defined ENDOR peaks are indeed ^2^H doublets associated with the −1/2 → +1/2 manifold, while signals from other manifolds are broadened so that they are indeed indistinguishable. The center frequency of the doublet seen at the edge of the EPR spectrum, 7.5 MHz (at 11 kG), is only slightly shifted from the ^2^H Larmor frequency ν_N_(^2^H) = 7.2 MHz and increases with field along with the Larmor frequency (Figure 5b), which indicates that the doublet is associated with the nominally −1/2 → +1/2 electron-spin transition (see Supporting Information). The overlaid EasySpin simulations (Figure 5b) show that this pattern is likewise well-replicated using the hyperfine tensor determined by simulating the low-field 2D pattern of Figure 5a.

### Calculations

To provide further electronic structure insights and also corroborate our experimental findings, (^tBu^**L**)MnI and (^tBu^**L**)MnH were subjected to computational analysis; tabulated spin-Hamiltonian parameters are presented in Table 1, along with the experimentally determined values for comparison (details are provided in the Supporting Information). The computed *g*-tensors for (^tBu^**L**)MnI and (^tBu^**L**)MnH exhibit little anisotropy, as expected for high-spin d^5^ centers. The predicted ZFS parameters are in reasonable agreement with experiment for (^tBu^**L**)MnH, and the calculated ^1^H hyperfine coupling tensor, both isotropic and anisotropic components, and spin density (ρ = −0.071) for the terminal hydride ligand of (^tBu^**L**)MnH are in excellent agreement with those determined by ENDOR spectroscopy.

**Table 1:** Collected Experimental and Calculated Spectroscopic Parameters for (^tBu^**L**)MnI and (^tBu^**L**)MnH

	(^tBu^L)MnI		(^tBu^L)MnH	
parameter	expt.	DFT	expt.	DFT
*g*	2.0^a^	[2.002, 2.009, 2.011]	2.0^a^	[2.001, 2.002, 2.002]
*D*(MHz; cm^–^^1^)	24,000; 0.83	105,000; 3.5	7600; 0.25	5850; 0.20
*E*/*D*	0.03	0.05	0.15	0.11
*A*(^55^Mn) (MHz)	–250^a^	–[88.9, 92.0, 92.9]	–250^a^	–[167, 176, 190]
*A*(^1^H) (MHz)			[−34.6, −34.6, 6.5]	[−34.2, −34.0, 7.4]

a Parameter assumed and not refined in EPR simulations.

The experimental ZFS parameters for (^tBu^**L**)MnI are not as well reproduced; for example, the absolute magnitude of *D* for (^tBu^**L**)MnI is about four times larger than the value given by the EPR simulation. Calculated values of |*D*| for Mn^2+^ complexes including heavy-element ligand(s) (e.g., I) can exhibit poor accuracy, presumably due to the approximate treatment of spin–orbit coupling under a scalar-relativistic Hamiltonian (see Supporting Information for further discussion).^65^ Nevertheless, the experimental trend is reproduced—i.e., (^tBu^**L**)MnI exhibits substantially higher *D* and much smaller rhombicity, *E*/*D*, compared to that of (^tBu^**L**)MnH. The results of this method accord well with our previous computational results on complexes of this ligand class,^54^ which demonstrated that spin densities computed at the TPSS0 level approximate those computed at the CASSCF level. A more consistent treatment of the ZFS would likely require a similar multireference ansatz to properly capture the effects of spin–orbit coupling.

## Discussion

The studies presented above provide the first characterization of a high-spin (*S* = 5/2), d^5^ metal hydride. Given the reasonable possibility that hydride-bound FeMoco intermediates feature locally d^5^ Fe^3+^–H sites, our work provides important context for the continued structural and electronic characterization of such catalytically relevant states. EPR/ENDOR studies of (^tBu^**L**)MnH/D reveal the bound hydride/deuteride to be, as expected, strongly coupled to the Mn^2+^ center (*a*_iso_(^1^H) = −20.9 MHz). The spectroscopic parameters determined for (^tBu^**L**)MnH/D are bolstered by DFT calculations, with which they are in excellent agreement. In line with all terminal hydride complexes for which such data are available, the anisotropic component of the Mn–H/D hyperfine tensor for (^tBu^**L**)MnH/D exhibits roughly axial symmetry.^17,18,73^ By contrast, this further cements the assignment of E_4_(4H)−which features a rhombic hyperfine-coupling tensor−as containing a Fe–H–Fe unit, rather than a terminally bound hydrido ligand(s).^10^ We note, however, that under catalytically relevant conditions, the Fe–H bonding is likely to be labile, with the hydride(s) potentially able to change coordination modes and/or migrate between different Fe sites within the cluster. Consequently, a terminal hydride(s) of Fe forming prior to reductive elimination of H_2_, as suggested by us elsewhere,^7,74,75^ may ultimately prove mechanistically important.

In terms of absolute magnitude, the ^1^H/^2^H hyperfine interactions observed for (^tBu^**L**)MnH/D are reasonably similar to those unambiguously determined for other half-integer spin terminal metal hydrides, irrespective of metal.^17,18,73,76^ Conspicuously, the value of *a*_iso_(^1^H) established for (^tBu^**L**)MnH appears to be considerably lower to that reported for low-spin (*S* = 1/2) [(dmpe)_2_MnH(L)]^+^ complexes (∼85 MHz; |ρ| ≈ 0.06).^53^ We emphasize, however, that ascertaining the ^1^H hyperfine couplings from analysis of the EPR spectra of the latter was compromised by the presence of extensive ^31^P hyperfine splittings and broadened lines. As such, these interactions could not be determined with precision; *a*_iso_(^1^H) for [(dmpe)_2_MnH(L)]^+^ could be as low as ∼40 MHz, which would be more usual for low-spin metal hydrides.^76^ Notably, (^tBu^**L**)MnH exhibits substantially more spin density on the hydrido ligand (|ρ| = 0.073; see above) than that of any other synthetic metal hydride for which *a*_iso_(^1^H) has been determined with reasonable accuracy. For example, the intermediate spin (*S* = 3/2) [(NacNac)Fe^+^H]^−^ (NacNac = β-diketiminate) exhibits |ρ| = 0.04,^18^ and |ρ| < 0.03 is typical.^16−20,73,76^ Direct comparison of spin densities for mononuclear species, such as (^tBu^**L**)MnH, and polynuclear systems, including FeMoco and its derivatives, requires knowledge of the spin-projection factors for the metal ions within the spin-coupled cluster assembly. Analysis of the hyperfine tensors for the Fe–H sites in the E_4_(4H) intermediate yields good estimates for the ratios of the spin-projection factors for the anchor Fe ions but not their absolute magnitudes.^11^

## Conclusions

Through the use of a sufficiently sterically demanding and σ-donating heteroscorpionate supporting ligand, we have isolated a complex with a hydride terminally bound to high-spin (*S* = 5/2) Mn^2+^, (^tBu^**L**)MnH. EPR and ENDOR analyses reveal an exceptional spin density on the Mn-bound hydride ligand, which is well-corroborated by DFT calculations. Given that hydride-bound FeMoco intermediates may feature locally high-spin Fe^3+^–H sites and the isoelectronic relationship between Mn^2+^ and Fe^3+^, our results further inform our understanding of such biological clusters. Future work will, quite naturally, aim to extend our Mn chemistry to Fe. We are curious to assess the extent to which the hydride chemistries of these metals substantially agree, where they deviate, and the implications of these results for nitrogenase enzymes.

## Supporting Information Available

Coordinates for all calculated structures (XYZ)
Spectroscopic data, additional figures, and discussion (PDF)

## Supplementary Material

ja4c03310_si_001.xyz

## References

[ref1] Norton J. R.; Sowa J. Introduction: Metal Hydrides. Chem. Rev. 2016, 116 (15), 8315–8317. 10.1021/acs.chemrev.6b00441. 27506870

[ref2] Crabtree R. H., The Organometallic Chemistry of the Transition Metals, 7th ed.; Wiley: Hoboken, N.J., 2019; pp 80–83.

[ref3] Can M.; Armstrong F. A.; Ragsdale S. W. Structure, Function, and Mechanism of the Nickel Metalloenzymes, CO Dehydrogenase, and Acetyl-CoA Synthase. Chem. Rev. 2014, 114 (8), 4149–4174. 10.1021/cr400461p. 24521136 PMC4002135

[ref4] Lubitz W.; Ogata H.; Rudiger O.; Reijerse E. Hydrogenases. Chem. Rev. 2014, 114 (8), 4081–4148. 10.1021/cr4005814. 24655035

[ref5] Hoffman B. M.; Lukoyanov D.; Yang Z. Y.; Dean D. R.; Seefeldt L. C. Mechanism of Nitrogen Fixation by Nitrogenase: The Next Stage. Chem. Rev. 2014, 114 (8), 4041–4062. 10.1021/cr400641x. 24467365 PMC4012840

[ref6] Schilter D.; Camara J. M.; Huynh M. T.; Hammes-Schiffer S.; Rauchfuss T. B. Hydrogenase Enzymes and Their Synthetic Models: The Role of Metal Hydrides. Chem. Rev. 2016, 116 (15), 8693–8749. 10.1021/acs.chemrev.6b00180. 27353631 PMC5026416

[ref7] Seefeldt L. C.; Yang Z. Y.; Lukoyanov D. A.; Harris D. F.; Dean D. R.; Raugei S.; Hoffman B. M. Reduction of Substrates by Nitrogenases. Chem. Rev. 2020, 120 (12), 5082–5106. 10.1021/acs.chemrev.9b00556. 32176472 PMC7703680

[ref8] Canfield D. E.; Glazer A. N.; Falkowski P. G. The Evolution and Future of Earth’s Nitrogen Cycle. Science 2010, 330 (6001), 192–196. 10.1126/science.1186120. 20929768

[ref9] Erisman J. W.; Sutton M. A.; Galloway J.; Klimont Z.; Winiwarter W. How a Century of Ammonia Synthesis Changed the World. Nat. Geosci. 2008, 1 (10), 636–639. 10.1038/ngeo325.

[ref10] Hoeke V.; Tociu L.; Case D. A.; Seefeldt L. C.; Raugei S.; Hoffman B. M. High-Resolution ENDOR Spectroscopy Combined with Quantum Chemical Calculations Reveals the Structure of Nitrogenase Janus Intermediate E_4_(4H). J. Am. Chem. Soc. 2019, 141 (50), 19950 10.1021/jacs.9b13035. 31310109 PMC6956989

[ref11] Igarashi R. Y.; Laryukhin M.; Dos Santos P. C.; Lee H. I.; Dean D. R.; Seefeldt L. C.; Hoffman B. M. Trapping H- Bound to the Nitrogenase FeMo-Cofactor Active Site During H_2_ Evolution: Characterization by ENDOR Spectroscopy. J. Am. Chem. Soc. 2005, 127 (17), 6231–6241. 10.1021/ja043596p. 15853328

[ref12] Lukoyanov D.; Khadka N.; Yang Z. Y.; Dean D. R.; Seefeldt L. C.; Hoffman B. M. Reductive Elimination of H_2_ Activates Nitrogenase to Reduce the N-N Triple Bond: Characterization of the E_4_(4H) Janus Intermediate in Wild-Type Enzyme. J. Am. Chem. Soc. 2016, 138 (33), 10674–10683. 10.1021/jacs.6b06362. 27529724 PMC5024552

[ref13] Lukoyanov D.; Yang Z. Y.; Dean D. R.; Seefeldt L. C.; Hoffman B. M. Is Mo Involved in Hydride Binding by the Four-Electron Reduced (E_4_) Intermediate of the Nitrogenase MoFe Protein?. J. Am. Chem. Soc. 2010, 132 (8), 2526–2527. 10.1021/ja910613m. 20121157 PMC2828500

[ref14] Lukoyanov D.; Khadka N.; Yang Z. Y.; Dean D. R.; Seefeldt L. C.; Hoffman B. M. Reversible Photoinduced Reductive Elimination of H_2_ from the Nitrogenase Dihydride State, the E_4_(4H) Janus Intermediate. J. Am. Chem. Soc. 2016, 138 (4), 1320–1327. 10.1021/jacs.5b11650. 26788586 PMC4773049

[ref15] Lukoyanov D. A.; Khadka N.; Yang Z. Y.; Dean D. R.; Seefeldt L. C.; Hoffman B. M. Hydride Conformers of the Nitrogenase FeMo-Cofactor Two-Electron Reduced State E_2_(2H), Assigned Using Cryogenic Intra Electron Paramagnetic Resonance Cavity Photolysis. Inorg. Chem. 2018, 57 (12), 6847–6852. 10.1021/acs.inorgchem.8b00271. 29575898 PMC6008734

[ref16] Keizer P. N.; Krusic P. J.; Morton J. R.; Preston K. F. Thiolato-Bridged and Selenato-Bridged Dinuclear Iron Carbonyl Radicals. J. Am. Chem. Soc. 1991, 113 (14), 5454–5456. 10.1021/ja00014a048.

[ref17] Kinney R. A.; Hetterscheid D. G. H.; Hanna B. S.; Schrock R. R.; Hoffman B. M. Formation of {[HIPTN_3_N]Mo(III)H} by Heterolytic Cleavage of H_2_ as Established by EPR and ENDOR Spectroscopy. Inorg. Chem. 2010, 49 (2), 704–713. 10.1021/ic902006v. 20000748 PMC2844792

[ref18] Chiang K. P.; Scarborough C. C.; Horitani M.; Lees N. S.; Ding K. Y.; Dugan T. R.; Brennessel W. W.; Bill E.; Hoffman B. M.; Holland P. L. Characterization of the Fe-H Bond in a Three-Coordinate Terminal Hydride Complex of Iron(I). Angew. Chem., Int. Ed. 2012, 51 (15), 3658–3662. 10.1002/anie.201109204. PMC3740144 22374689

[ref19] Kinney R. A.; Saouma C. T.; Peters J. C.; Hoffman B. M. Modeling the Signatures of Hydrides in Metalloenzymes: ENDOR Analysis of a Di-Iron Fe(μ-NH)(μ-H)Fe Core. J. Am. Chem. Soc. 2012, 134 (30), 12637–12647. 10.1021/ja303739g. 22823933 PMC3433054

[ref20] Arnett C. H.; Bogacz I.; Chatterjee R.; Yano J.; Oyala P. H.; Agapie T. Mixed-Valent Diiron μ-Carbyne, μ-Hydride Complexes: Implications for Nitrogenase. J. Am. Chem. Soc. 2020, 142 (44), 18795–18813. 10.1021/jacs.0c05920. 32976708

[ref21] Jewson J. D.; Liable-Sands L. M.; Yap G. P. A.; Rheingold A. L.; Theopold K. H. Paramagnetic Alkyl, Hydride, and Alkene Complexes of the Tp(tBu,Me)Co Moiety. Organometallics 1999, 18 (3), 300–305. 10.1021/om980844f.

[ref22] Smith J. M.; Lachicotte R. J.; Holland P. L. NN Bond Cleavage by a Low-Coordinate Iron(II) Hydride Complex. J. Am. Chem. Soc. 2003, 125 (51), 15752–15753. 10.1021/ja038152s. 14677959

[ref23] Sadique A. R.; Gregory E. A.; Brennessel W. W.; Holland P. L. Mechanistic Insight into NN Cleavage by a Low-Coordinate Iron(II) Hydride Complex. J. Am. Chem. Soc. 2007, 129 (26), 8112–8121. 10.1021/ja069199r. 17564444 PMC2548314

[ref24] Yu Y.; Sadique A. R.; Smith J. M.; Dugan T. R.; Cowley R. E.; Brennessel W. W.; Flaschenriem C. J.; Bill E.; Cundari T. R.; Holland P. L. The Reactivity Patterns of Low-Coordinate Iron-Hydride Complexes. J. Am. Chem. Soc. 2008, 130 (20), 6624–6638. 10.1021/ja710669w. 18444648 PMC2474859

[ref25] Ding K. Y.; Brennessel W. W.; Holland P. L. Three-Coordinate and Four-Coordinate Cobalt Hydride Complexes That React with Dinitrogen. J. Am. Chem. Soc. 2009, 131 (31), 10804–10805. 10.1021/ja902812y. 19621923 PMC2737074

[ref26] Blair V. L.; Carrella L. M.; Clegg W.; Klett J.; Mulvey R. E.; Rentschler E.; Russo L. Structural and Magnetic Insights into the Trinuclear Ferrocenophane and Unexpected Hydrido Inverse Crown Products of Alkali-Metal-Mediated Manganation(II) of Ferrocene. Chem.—Eur. J. 2009, 15 (4), 856–863. 10.1002/chem.200802086. 19105193

[ref27] Chomitz W. A.; Arnold J. Synthesis and Characterization of Manganese and Iron Complexes Supported by Multidentate [N_2_P_2_] Ligands. Dalton Trans. 2009, (10), 1714–1720. 10.1039/b821954k. 19240904

[ref28] Yao S. L.; Xiong Y.; Driess M. Facile Metalation of Silicon and Germanium Analogues of Thiocarboxylic Acids with a Manganese(II) Hydride Precursor. Chem.—Eur. J. 2012, 18 (36), 11356–11361. 10.1002/chem.201201335. 22829217

[ref29] Lee Y.; Anderton K. J.; Sloane F. T.; Ermert D. M.; Abboud K. A.; Garcia-Serres R.; Murray L. J. Reactivity of Hydride Bridges in High-Spin [3M-3(μ-H)] Clusters (M = Fe^II^, Co^II^). J. Am. Chem. Soc. 2015, 137 (33), 10610–10617. 10.1021/jacs.5b05204. 26270596

[ref30] Arnet N. A.; Dugan T. R.; Menges F. S.; Mercado B. Q.; Brennessel W. W.; Bill E.; Johnson M. A.; Holland P. L. Synthesis, Characterization, and Nitrogenase-Relevant Reactions of an Iron Sulfide Complex with a Bridging Hydride. J. Am. Chem. Soc. 2015, 137 (41), 13220–13223. 10.1021/jacs.5b06841. 26457740 PMC4818001

[ref31] Gehring H.; Metzinger R.; Braun B.; Herwig C.; Harder S.; Ray K.; Limberg C. An iron(ii) hydride complex of a ligand with two adjacent β-diketiminate binding sites and its reactivity. Dalton Trans. 2016, 45 (7), 2989–2996. 10.1039/C5DT04266F. 26757878 PMC5536248

[ref32] Bellows S. M.; Arnet N. A.; Gurubasavaraj P. M.; Brennessel W. W.; Bill E.; Cundari T. R.; Holland P. L. The Mechanism of N-N Double Bond Cleavage by an Iron(II) Hydride Complex. J. Am. Chem. Soc. 2016, 138 (37), 12112–12123. 10.1021/jacs.6b04654. 27598037 PMC5499983

[ref33] Fohlmeister L.; Jones C. Stabilisation of Carbonyl Free Amidinato-Manganese(II) Hydride Complexes: ″Masked″ Sources of Manganese(I) in Organometallic Synthesis. Dalton Trans. 2016, 45 (4), 1436–1442. 10.1039/C5DT04504E. 26674008

[ref34] Anderton K. J.; Ermert D. M.; Quintero P. A.; Turvey M. W.; Fataftah M. S.; Abboud K. A.; Meisel M. W.; Cizmar E.; Murray L. J. Correlating Bridging Ligand with Properties of Ligand-Templated [Mn^II^_3_X_3_]^3+^ Clusters (X = Br^–^, Cl^–^, H^–^, MeO^–^). Inorg. Chem. 2017, 56 (19), 12012–12022. 10.1021/acs.inorgchem.7b02004. 28920698

[ref35] Anderton K. J.; Knight B. J.; Rheingold A. L.; Abboud K. A.; Garcia-Serres R.; Murray L. J. Reactivity of Hydride Bridges in a High-Spin [Fe_3_(μ-H)_3_]_3+_ Cluster: Reversible H_2_/CO Exchange and Fe-H/B-F Bond Metathesis. Chem. Sci. 2017, 8 (5), 4123–4129. 10.1039/C6SC05583D. 28603601 PMC5443887

[ref36] MacLeod K. C.; Lewis R. A.; DeRosha D. E.; Mercado B. Q.; Holland P. L. C-H and C-N Activation at Redox-Active Pyridine Complexes of Iron. Angew. Chem., Int. Ed. 2017, 56 (4), 1069–1072. 10.1002/anie.201610679. PMC5266524 28000416

[ref37] Sekiguchi Y.; Kuriyama S.; Eizawa A.; Arashiba K.; Nakajima K.; Nishibayashi Y. Synthesis and Reactivity of Iron-Dinitrogen Complexes Bearing Anionic Methyl- and Phenyl-Substituted Pyrrole-Based PNP-Type Pincer Ligands Toward Catalytic Nitrogen Fixation. Chem. Commun. 2017, 53 (88), 12040–12043. 10.1039/C7CC06987A. 29063100

[ref38] Hein N. M.; Pick F. S.; Fryzuk M. D. Synthesis and Reactivity of a Low-Coordinate Iron(II) Hydride Complex: Applications in Catalytic Hydrodefluorination. Inorg. Chem. 2017, 56 (23), 14513–14523. 10.1021/acs.inorgchem.7b02199. 29144749

[ref39] Ott J. C.; Wadepohl H.; Enders M.; Gade L. H. Taking Solution Proton NMR to Its Extreme: Prediction and Detection of a Hydride Resonance in an Intermediate-Spin Iron Complex. J. Am. Chem. Soc. 2018, 140 (50), 17413–17417. 10.1021/jacs.8b11330. 30486649

[ref40] Hickey A. K.; Greer S. M.; Valdez-Moreira J. A.; Lutz S. A.; Pink M.; DeGayner J. A.; Harris T. D.; Hill S.; Telser J.; Smith J. M. A Dimeric Hydride-Bridged Complex with Geometrically Distinct Iron Centers Giving Rise to an *S* = 3 Ground State. J. Am. Chem. Soc. 2019, 141 (30), 11970–11975. 10.1021/jacs.9b04389. 31283232

[ref41] Gasperini D.; King A. K.; Coles N. T.; Mahon M. F.; Webster R. L. Seeking Heteroatom-Rich Compounds: Synthetic and Mechanistic Studies into Iron Catalyzed Dehydrocoupling of Silanes. ACS Catal. 2020, 10 (11), 6102–6112. 10.1021/acscatal.0c01440.

[ref42] McWilliams S. F.; Broere D. L. J.; Halliday C. J. V.; Bhutto S. M.; Mercado B. Q.; Holland P. L. Coupling Dinitrogen and Hydrocarbons through Aryl Migration. Nature 2020, 584 (7820), 221–226. 10.1038/s41586-020-2565-5. 32788733 PMC7430000

[ref43] Handford R. C.; Nguyen T. T.; Teat S. J.; Britt R. D.; Tilley T. D. Direct Transformation of SiH_4_ to a Molecular L(H)Co = Si = Co(H)_2_L Silicide Complex. J. Am. Chem. Soc. 2023, 145, 3031–3039. 10.1021/jacs.2c11569. 36696099

[ref44] McWilliams S. F.; Mercado B. Q.; MacLeod K. C.; Fataftah M. S.; Tarrago M.; Wang X. P.; Bill E.; Ye S. F.; Holland P. L. Dynamic Effects on Ligand Field from Rapid Hydride Motion in an Iron(II) Dimer with an *S* = 3 Ground State. Chem. Sci. 2023, 14 (9), 2303–2312. 10.1039/D2SC06412J. 36873832 PMC9977447

[ref45] Van Stappen C.; Decamps L.; Cutsail G. E.; Bjornsson R.; Henthorn J. T.; Birrell J. A.; DeBeer S. The Spectroscopy of Nitrogenases. Chem. Rev. 2020, 120 (12), 5005–5081. 10.1021/acs.chemrev.9b00650. 32237739 PMC7318057

[ref46] Beinert H.; Holm R. H.; Münck E. Iron-Sulfur Clusters: Nature’s Modular, Multipurpose Structures. Science 1997, 277 (5326), 653–659. 10.1126/science.277.5326.653. 9235882

[ref47] Ye M.; Thompson N. B.; Brown A. C.; Suess D. L. M. A Synthetic Model of Enzymatic [Fe_4_S_4_]-Alkyl Intermediates. J. Am. Chem. Soc. 2019, 141 (34), 13330–13335. 10.1021/jacs.9b06975. 31373801 PMC6748666

[ref48] McSkimming A.; Sridharan A.; Thompson N. B.; Muller P.; Suess D. L. M. An [Fe_4_S_4_]^3+^-Alkyl Cluster Stabilized by an Expanded Scorpionate Ligand. J. Am. Chem. Soc. 2020, 142 (33), 14314–14323. 10.1021/jacs.0c06334. 32692919 PMC7442740

[ref49] Sridharan A.; Brown A. C.; Suess D. L. M. A Terminal Imido Complex of an Iron-Sulfur Cluster. Angew. Chem., Int. Ed. 2021, 60 (23), 12802–12806. 10.1002/anie.202102603. PMC8294340 33772994

[ref50] Brown A. C.; Thompson N. B.; Suess D. L. M. Evidence for Low-Valent Electronic Configurations in Iron-Sulfur Clusters. J. Am. Chem. Soc. 2022, 144 (20), 9066–9073. 10.1021/jacs.2c01872. 35575703

[ref51] Kim Y.; Sridharan A.; Suess D. L. M. The Elusive Mononitrosylated [Fe_4_S_4_] Cluster in Three Redox States. Angew. Chem., Int. Ed. 2022, 61 (47), e202213032 10.1002/anie.202213032. PMC9669169 36194444

[ref52] Maekawa M.; Römelt M.; Daniliuc C. G.; Jones P. G.; White P. S.; Neese F.; Walter M. D. Reactivity studies on [Cp′MnX(thf)]2: manganese amide and polyhydride synthesis. Chem. Sci. 2012, 3 (10), 2972–2979. 10.1039/c2sc20737k.

[ref53] Rennie B. E.; Price J. S.; Emslie D. J. H.; Morris R. H. Trans Ligand Determines the Stability of Paramagnetic Manganese(II) Hydrides of the Type *trans*-[MnH(L)(dmpe)_2_]^+^ Where L is PMe_3_, C_2_H_4_, or CO. Inorg. Chem. 2023, 62 (21), 8123–8135. 10.1021/acs.inorgchem.2c04432. 36812512

[ref54] McSkimming A.; Thompson N. B. Four-Coordinate Fe N_2_ and Imido Complexes Supported by a Hemilabile NNC Heteroscorpionate Ligand. Inorg. Chem. 2022, 61 (31), 12318–12326. 10.1021/acs.inorgchem.2c01656. 35895990 PMC9367695

[ref55] Gu L.; Fraker A.; McSkimming A. Dynamic N_2_ Binding at High-Spin Co(I) Supported by N,N,C Heteroscorpionates. Organometallics 2023, 42 (13), 1621–1628. 10.1021/acs.organomet.3c00183.

[ref56] Kaesz H. D.; Saillant R. B. Hydride complexes of the transition metals. Chem. Rev. 1972, 72 (3), 231–281. 10.1021/cr60277a003.

[ref57] Stoll S.; Schweiger A. Easyspin, a Comprehensive Software Package for Spectral Simulation and Analysis in EPR. J. Magn. Reson. 2006, 178 (1), 42–55. 10.1016/j.jmr.2005.08.013. 16188474

[ref58] Smoukov S. K.; Telser J.; Bernat B. A.; Rife C. L.; Armstrong R. N.; Hoffman B. M. EPR Study of Substrate Binding to the Mn(II) Active Site of the Bacterial Antibiotic Resistance Enzyme Fosa: A Better Way to Examine Mn(II). J. Am. Chem. Soc. 2002, 124 (10), 2318–2326. 10.1021/ja012480f. 11878987

[ref59] Duboc C.; Collomb M. N.; Neese F. Understanding the Zero-Field Splitting of Mononuclear Manganese(II) Complexes from Combined EPR Spectroscopy and Quantum Chemistry. Appl. Magn. Reson. 2010, 37 (1–4), 229–245. 10.1007/s00723-009-0085-4.

[ref60] Sharma A.; Gaidamakova E. K.; Matrosova V. Y.; Bennett B.; Daly M. J.; Hoffman B. M. Responses of Mn^2+^speciation in*Deinococcus radiodurans*and*Escherichia coli*to γ-radiation by advanced paramagnetic resonance methods. Proc. Natl. Acad. Sci. U.S.A. 2013, 110 (15), 5945–5950. 10.1073/pnas.1303376110. 23536297 PMC3625348

[ref61] Wood R. M.; Stucker D. M.; Jones L. M.; Lynch W. B.; Misra S. K.; Freed J. H. An EPR Study of Some Highly Distorted Tetrahedral Manganese(II) Complexes at High Magnetic Fields. Inorg. Chem. 1999, 38 (23), 5384–5388. 10.1021/ic990377+.

[ref62] Girolami G. S.; Wilkinson G.; Galas A. M. R.; Thorntonpett M.; Hursthouse M. B. Synthesis and Properties of the Divalent 1,2-Bis(Dimethylphosphino)Ethane (Dmpe) Complexes MCl_2_(dmpe)_2_ and MMe_2_(dmpe)_2_ (M = Ti, V, Cr, Mn, or Fe) - X-Ray Crystal-Structures of MCl_2_(dmpe)_2_ (M = Ti, V, or Cr), MnBr_2_(dmpe)_2_, TiMe_1.3_Cl_0.7_(dmpe)_2_, and CrMe_2_(dmpe). J. Chem. Soc., Dalton Trans. 1985, (7), 1339–1348. 10.1039/dt9850001339.

[ref63] Mantel C.; Baffert C.; Romero I.; Deronzier A.; Pécaut J.; Collomb M. N.; Duboc C. Structural Characterization and Electronic Properties Determination by High-Field and High-Frequency EPR of a Series of Five-Coordinated Mn(II) Complexes. Inorg. Chem. 2004, 43 (20), 6455–6463. 10.1021/ic049650k. 15446897

[ref64] Duboc C.; Phoeung T.; Zein S.; Pecaut J.; Collomb M. N.; Neese F. Origin of the Zero-Field Splitting in Mononuclear Octahedral Dihalide Mn^II^ Complexes: An Investigation by Multifrequency High-Field Electron Paramagnetic Resonance and Density Functional Theory. Inorg. Chem. 2007, 46 (12), 4905–4916. 10.1021/ic062384l. 17508742

[ref65] Zein S.; Duboc C.; Lubitz W.; Neese F. A Systematic Density Functional Study of the Zero-Field Splitting in Mn(II) Coordination Compounds. Inorg. Chem. 2008, 47 (1), 134–142. 10.1021/ic701293n. 18072763

[ref66] Horitani M.; Offenbacher A. R.; Carr C. A. M.; Yu T.; Hoeke V.; Cutsail G. E.; Hammes-Schiffer S.; Klinman J. P.; Hoffman B. M. ^13^C ENDOR Spectroscopy of Lipoxygenase–Substrate Complexes Reveals the Structural Basis for C–H Activation by Tunneling. J. Am. Chem. Soc. 2017, 139 (5), 1984–1997. 10.1021/jacs.6b11856. 28121140 PMC5322796

[ref67] Sharma A.; Whittington C.; Jabed M.; Hill S. G.; Kostenko A.; Yu T.; Li P. F.; Doan P. E.; Hoffman B. M.; Offenbacher A. R. ^13^C Electron Nuclear Double Resonance Spectroscopy-Guided Molecular Dynamics Computations Reveal the Structure of the Enzyme-Substrate Complex of an Active, N-Linked Glycosylated Lipoxygenase. Biochemistry 2023, 62 (10), 1531–1543. 10.1021/acs.biochem.3c00119. 37115010 PMC10704959

[ref68] Schweiger A.; Jeschke G. Principles of Pulse Electron Paramagnetic Resonance; Oxford University Press: Oxford, UK, 2001; pp 1–578.

[ref69] Doan P. E.; Hoffman B. M. Making Hyperfine Selection in Mims ENDOR Independent of Deadtime. Chem. Phys. Lett. 1997, 269 (3–4), 208–214. 10.1016/S0009-2614(97)00293-5.

[ref70] Astashkin A. V.; Raitsimring A. M. Refocused Primary Echo: A Zero Dead Time Detection of the Electron Spin Echo Envelope Modulation. J. Magn. Reson. 2000, 143 (2), 280–291. 10.1006/jmre.1999.1988. 10729254

[ref71] Snetsinger P. A.; Chasteen N. D.; Vanwilligen H. Structural-Analysis of a Low-Spin Cyanide Adduct of Iron(III) Transferrin by Angle-Selected ^13^C ENDOR Spectroscopy. J. Am. Chem. Soc. 1990, 112 (22), 8155–8160. 10.1021/ja00178a046.

[ref72] Fitzpatrick J. A. J.; Manby F. R.; Western C. M. The Interpretation of Molecular Magnetic Hyperfine Interactions. J. Chem. Phys. 2005, 122 (8), 084312 10.1063/1.1851501. 15836044

[ref73] Gu N. X.; Oyala P. H.; Peters J. C. H_2_ Evolution from a Thiolate-Bound Ni(III) Hydride. J. Am. Chem. Soc. 2020, 142 (17), 7827–7835. 10.1021/jacs.0c00712. 32249575

[ref74] Raugei S.; Seefeldt L. C.; Hoffman B. M. Critical Computational Analysis Illuminates the Reductive-Elimination Mechanism That Activates Nitrogenase for N_2_ Reduction. Proc. Natl. Acad. Sci. U.S.A. 2018, 115 (45), E10521–E10530. 10.1073/pnas.1810211115. 30355772 PMC6233137

[ref75] Lukoyanov D. A.; Yang Z. Y.; Dean D. R.; Seefeldt L. C.; Raugei S.; Hoffman B. M. Electron Redistribution within the Nitrogenase Active Site FeMo-Cofactor During Reductive Elimination of H_2_ to Achieve N≡N Triple-Bond Activation. J. Am. Chem. Soc. 2020, 142 (52), 21679–21690. 10.1021/jacs.0c07914. 33326225 PMC7783777

[ref76] Hu Y.; Shaw A. P.; Estes D. P.; Norton J. R. Transition-Metal Hydride Radical Cations. Chem. Rev. 2016, 116 (15), 8427–8462. 10.1021/acs.chemrev.5b00532. 26828562

