# Correction to “Terminal Hydride Complex of High-Spin Mn”

**DOI:** 10.1021/jacs.4c11609

**Published:** 2024-09-18

**Authors:** Alex Drena, Addison Fraker, Niklas B. Thompson, Peter E. Doan, Brian M. Hoffman, Alex McSkimming

The following statement was omitted from the Acknowledgments:

