# Supplementary material for: Terminal Hydride Complex of High-Spin Mn
Source: J Am Chem Soc. 2024 Jun 28;146(27):18370–8. doi: 10.1021/jacs.4c03310 (PMC11240256; doi:10.1021/jacs.4c03310)
Supplement: Supplementary file 2 — ja4c03310_si_002.pdf [file ja4c03310_si_002.pdf]

Supporting Information for:

A Terminal Hydride Complex of High-spin Mn

Alex Drena,[2]† Addison Fraker,[1]† Niklas B. Thompson,[3] Peter E. Doan,[2] Brian M. Hoffman,[2]* Alex McSkimming,[1]**

[1]Department of Chemistry, Tulane University, New Orleans, LA 70118; [2]Department of Chemistry, Northwestern University, Evanston, IL 60208; [3]Chemical Sciences and Engineering Division, Argonne National Laboratory, Lemont, IL 60439.

*bmh@northwestern.edu

**amcskimming@tulane.edu

† Signifies equal contributions.

Contents

Experimental	S3
Spectroscopic Data	S7
Additional Data and Discussions	
Calculations	S15
EPR Spectroscopy	S18
References	S29

Experimental

General Methods. All manipulations involving metal complexes were carried out in an N_2 atmosphere glovebox. Glassware was oven-dried for at least several hours at 160 °C prior to use.

Materials. All solvents except *n*-pentane and 1,2-difluorobenzene (DFB) were distilled from purple Na/benzophenone prior to use. All solvents were stored over activated 3 Å molecular sieves for at least 24 h prior to use. All reagents were purchased from commercial suppliers and used without further purification unless otherwise noted. ^{tBu}**L**H was prepared according to literature procedures.[1]

Comment on purity. Organic products were deemed pure by ^{1}H NMR spectroscopy. All metal complexes were isolated as crystalline solids that were homogenous under microscope inspection. The purity of metal complexes was readily assessed by a combination of ^{1}H and ^{19}F NMR spectroscopies. The latter proved particularly useful, as the paramagnetism of (^{tBu}**L**)MnI and (^{tBu}**L**)MnH rendered thorough interpretation of their highly broadened ^{1}H NMR spectra difficult. The total integrated area for impurity peaks constituted <3% of the ^{19}F content for the Mn complexes. Both Mn complexes formed homogenous solutions in non-coordinating solvents, *e.g.* C_6H_6, and were ^{7}Li NMR silent, thus precluding the presence of inorganic salts. We have also obtained elemental analyses for the Mn complexes, with results given below. These conform to the expected compositions.

Spectroscopy and Spectrometry. NMR spectra were recorded on a Bruker 400 MHz spectrometer. ^{1}H and ^{13}C chemical shifts are reported in ppm relative to tetramethylsilane using residual solvent as an internal standard. ^{19}F chemical shifts are reported in ppm relative to 5% v/v internal PhF[2] and ^{11}B chemical shifts relative to $BF_3.Et_2O$ (capillary). Solution-phase effective magnetic moments were determined by the method described by Evans[3] and are corrected for diamagnetic contributions.[4] Mass spectrometry data were collected on a Bruker micrOTOF II with an ESI source. FTIR spectra were recorded on solid or solution samples using a Bruker Alpha II FTIR spectrometer operating at 4 cm^{-1} resolution. Elemental analyses were collected by Midwest Microlabs.

X-Ray Crystallography. Low-temperature diffraction data were collected on a Bruker-AXS X8 Kappa Duo diffractometer coupled to an APEX2 CCD detector. The data collections were executed with Mo K_α radiation (λ = 0.71073 Å) from a *IμS* micro-source, performing ϕ-and ω-scans. Absorption and other corrections were applied using the program SADABS.[5,6] The structures were solved by dual-space methods using SHELXT[7] and refined against F^2 on all data by full-matrix least squares with SHELXL-2017,[8] following established refinement strategies.[9] All non-hydrogen atoms were refined anisotropically, and all hydrogen atoms were included into the model at geometrically calculated positions and refined using a riding model.

Computational details. Density functional theory calculations carried out using revision 5.0.3 of the ORCA suite of programs,[10] using the 'TIGHTSCF' convergence criteria and default settings otherwise. Given our previous experience with complexes of the '^{R}**L**' class of ligands,[1] all DFT calculations made use of the TPSS meta-GGA exchange-correlation functional, including either 0% (TPSS), 10% (TPSSh), or 25% (TPSS0) exact Hartree-Fock (HF) exchange.[11-13] Scalar relativistic effects were included using the zeroth order regular approximation (ZORA),[14] while the effects of dispersion were treated according to the Becke-Johnson damping scheme (D3BJ) of Grimme and co-workers.[15-16] Initial geometries for (^{tBu}**L**)MnI and (^{tBu}**L**)MnH were taken from the crystallographically-determined coordinates. These geometries were subsequently optimized employing the re-contracted ZORA-def2-TZVP basis for all atoms heavier than C (SARC-def2-TZVP for I), the C(H) moiety directly bound to the Mn center, and the hydride in the case of (^{tBu}**L**)MnH; the smaller ZORA-def2-SVP basis was used for all other C and H atoms.[17-18] For all atoms, the general-purpose segmented all-electron relativistically-contracted auxiliary Coulomb fitting basis (SARC/J) was employed, which is a decontraction of the def2/J basis developed by Weigend.[19] Calculations including HF exchange were accelerated through the RIJCOSX approximation.[20]

EPR Spectroscopy. 35 GHz CW EPR spectra of the S = 5/2 Mn^{2+} complexes were collected at ~2 K on a modified Varian E-110 spectrometer.[21] Simulation of such EPR spectra using EasySpin[22] involve input of ZFS parameters, D and E, and a distribution parameter representing a Gaussian distribution in D and E with breadth as a fraction (f) of each parameter, as well as ^{55}Mn hyperfine coupling, A.[23] 35 GHz 2K pulses ENDOR measurements employed a spectrometer described previously,[24] with SpinCore PulseBlaster ESR_PRO 400 MHz digital word generator and Agilent Technologies Acquiris DP235 500 MS/s digitizer using SpecMan4EPR software.[25] Davies ENDOR employs a pulse sequence (π−T_{rf}−π/2−τ−π−τ-echo, where T_{rf} is the rf pulse); refocused-Mims (ReMIMS) employing a 4-pulse sequence (π/2−τ−π/2−T_{rf}−π/2−τ_2−π−τ_2−echo, with rf pulse T_{rf}) was used to study τ-dependence of the ^{2}H hyperfine coupling. The ReMIMS ENDOR response (R) follows the same periodic dependence on the hyperfine coupling constant, A, and τ as Mims ENDOR: $R \propto \left[1 - \cos(2\pi A\tau)\right]$.

Synthetic Procedures. KEt_3BD. To a stirred suspension of LiD (0.065 g, 7.3 mmol) in THF (6 mL) was added Et_3B (1.0 M in hexanes, 8 mL, 8 mmol) via syringe (no real care is required here, as the reaction is slow). After stirring at RT for 18h volatiles were removed *thoroughly* under reduced pressure (to an essentially constant mass), leaving a colorless oil. Pentane (10mL) was added to the oil (in which it has no apparent solubility). To this mixture solid tBuOK (0.818 g, 7.29 mmol) was added cautiously via spatula (*slight exotherm*) and stirring continued overnight. The minimum benzene (~ 2 mL) was then added to fully dissolve the deposited colorless oil and a small amount of insoluble residue removed by filtration through a short pad of Celite. Volatiles were removed under reduced pressure, leaving a colorless oil.

Pentane (5 mL) was added and the mixture stirred rapidly for several hours to afford an oily, colorless solid and a colorless supernatant. The solid was collected on a 10 mL glass fritted funnel, washed with pentane (3 × 3 mL), and N_2 from the box atmosphere was pulled through the material for several minutes. This procedure serves to mechanically separate the bulk of the oily material from the desired solid. The solid was re-dissolved in benzene and the solvent removed under reduced pressure. Pentane (5 mL) was again added to the resulting oily solid with stirring, which was again collected on a frit and washed with pentane (3 × 3 mL). Dissolving the solid in benzene, filtering through a small pad of Celite and removing all volatiles affords KEt_3BD as a free-flowing, colorless solid. Yield: 0.407 g (41 % over two steps). Although KEt_3BD has been previously reported,[26] spectroscopic data was not provided. ^1H NMR (300 MHz, C_6D_6) δ 1.11 (3H, t, J = 7 Hz, $BCH_2C\mathit{H}_3$), 0.20 (3H, t, J = 7 Hz, $BC\mathit{H}_2CH_3$). ^{11}B NMR (96 MHz, C_6D_6) δ –13.89. FTIR cm^{-1}: see Figure S3.

(^{tBu}L)MnI. A solution of ^{tBu}**L** (2.16 g, 3.29 mmol) in THF (25 mL) was cooled to –78 °C in the glove box cold well. A solution of tBuLi in hexanes (2.7 M, 1.34 mL, 3.62 mmol) was added dropwise via syringe with stirring and the resulting solution placed in the glovebox freezer at –30 °C (stirring is not necessary at this stage). After 2h, solid $MnI_2(THF)_3$ (2.06 g, 4.55 mmol) was added to the cold mixture with stirring. The suspension was allowed to warm to room temperature (RT) and stirred for an additional hour to afford a brown-yellow solution. Volatiles were removed under reduced pressure to yield a viscous yellow oil. Pentane (15 mL) was added, which caused the oil to rapidly solidify into a yellow mass. The pentane was decanted from the crude solid, which was washed with additional pentane (2 × 10 mL). The crude material was then suspended in Et_2O (6 mL) and stirred vigorously for 15 mins to dissolve LiI. The resulting yellow solid was collected on a 10 mL glass fritted funnel and washed with Et_2O (3 × 3 mL) and pentane (2 × 3 mL). The crude material was dissolved in THF (20 mL), diluted with an equal volume of pentane and the solution run through 6 cm pad of silica on a 30 mL glass fritted funnel (this process removes residual $MnI_2(THF)_3$). The silica was washed with a 1:1 mixture of THF-pentane until the washings were colorless. The success of these operations could be assessed by removing the SiO_2 pad from the glovebox and allowing adsorbed material to be oxidized; the lower half of the SiO_2 should remain entirely colorless. Volatiles were removed from the collected fractions under reduced pressure to yield a yellow crystalline solid. The solid was suspended in Et_2O (6 mL) and pentane (30 mL) added to complete crystallization. The pale-yellow, crystalline (^{tBu}**L**)MnI was collected on a 10 mL glass fritted funnel and washed with pentane (3 × 5 mL). Yield: 1.76 g (64 %). X-ray quality crystals were grown by cooling a saturated Et_2O solution of the complex to –30 °C overnight. RT magnetic moment (by Evans method in C_6D_6): 6.1 μ_B. ^1H NMR (400 MHz, C_6D_6) δ 62.03, 4.26, 1.93, –72.04. ^{19}F NMR (376 MHz, C_6D_6) δ –84.76 (s, $C\mathit{F}_3$). FTIR cm^{-1}: 2963m, 2876m, 2362w, 2164w, 2000w, 1942w, 1595w, 1537w, 1525w, 1450m, 1416w, 1364s, 1291m, 1242s, 1160m, 1121w, 1097s, 1042s, 993w, 946m, 878w, 851m, 827m, 813m, 786w, 744w, 728w, 702w,

680w, 652w, 614w, 561w, 477w, 436w. UV–vis (C_6H_6) λ_{max} (nm) ε_{max} (cm^{-1} M^{-1}): 420 (sh), 306 (1.2×10^3). Samples for EPR and elemental analyses were obtained as the C_6H_6 solvate by recrystallization from C_6H_6-pentane. The amount of C_6H_6 (1.25 equiv.) was quantified by ^{1}H NMR spectroscopy using 1,3,5-trimethoxybenzene as an internal standard. Anal. Calc. for $C_{34}H_{49}F_6MnN_4SiI$•1.25(C_6H_6): C 53.29; H 6.09; N 5.99. Found: C 53.23; H 6.39; N 6.13.

(^{tBu}L)MnH. A solution of (^{tBu}**L**)MnI (300 mg, 0.358 mmol) in 5 mL DFB was cooled in the glovebox freezer to –30 °C. This solution was removed from the freezer and a solution of KEt_3BH (0.074 g, 0.54 mmol) in DFB (1 mL) was added dropwise to the still cold reaction mixture resulting in appreciable darkening and rapid precipitation of KI. The reasonably large excess of K[Et_3BH] is necessary to ensure full conversion of the starting (^{tBu}**L**)MnI complex and does not affect the final purity of (^{tBu}**L**)MnH. The suspension was allowed to come to RT and filtered through a short pad (~1 cm) of Celite in glass pipette. Volatiles were removed under reduced pressure leaving a yellow oily residue. ((Me_3)$_3$SiO)$_2$ (~2 mL) was added and the suspension rapidly stirred for ~15 mins to afford a yellow solid with a brown supernatant. The crude solid was collected on a pad of Celite and washed with additional ((Me_3)$_3$SiO)$_2$ (3 × 1 mL). This solid was mostly (^{tBu}**L**)MnH by NMR analysis, but contaminated with diamagnetic, B-containing impurities. The latter were removed by dissolving the solid in the minimum Et_2O (~10 mL), concentrating to ~2 mL and storing the solution at –30 °C overnight. This affords the product as bright yellow crystals. These crystals were suitable for XRD studies. The supernatant was decanted and the crystals washed with additional cold (–30 °C) Et_2O (3 × 1 mL). Finally, this material was recrystallized by dissolving in the minimum C_6H_6, diluting with 4 volumes of pentane and standing at –30 °C overnight to give large yellow blocks of (^{tBu}**L**)MnH as the pentane monosolvate. Yield: 107 mg (38 %). (^{tBu}**L**)MnD was made similarly from KEt_3BD. RT magnetic moment (by Evans method in C_6D_6): 6.0 μ_B. ^{1}H NMR (400 MHz, C_6D_6) δ 9.72, 4.82, 2.81. ^{19}F NMR (376 MHz, C_6D_6): δ −79.91 (s, CF_3). FTIR cm^{-1}: 3848w, 3740w, 3624w, 2962m, 2904w, 2870w, 2627w, 2359w, 2333w, 2207w, 2176w, 2137w, 2060w, 2037w, 2002w, 1953w, 1588m (Mn–H), 1537m, 1524m, 1461w, 1444w, 1416w, 1364s, 1290w, 1272s, 1243m, 1218w, 1205w, 1161s, 1122s, 1096w, 1057w, 1023w, 992w, 947m, 875w, 850w, 826w, 811w, 784w, 770w, 742w, 729w, 702w, 680w, 667w, 651w, 630w, 614w, 560w, 476w, 453w, 430w. UV–vis (C_6H_6) λ_{max} (nm) ε_{max} (cm^{-1} M^{-1}): 432 (sh), 317 (1.1×10^3). Anal. Calc. for $C_{34}H_{50}F_6MnN_4Si$•C_5H_{12}: C 59.75; H 7.97; N 7.15. Found: C 59.72; H 7.73; N 7.14.

Spectroscopic Data

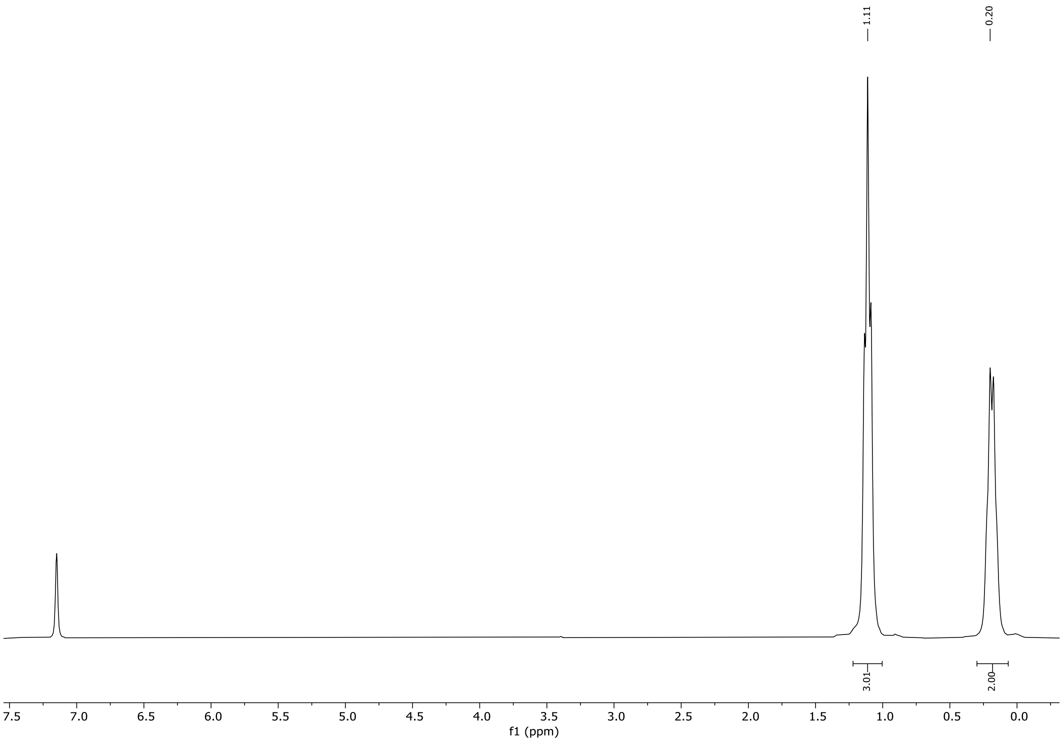

Figure S1. ^{1}H NMR spectrum of $KBEt_3D$ in C_6D_6 at 300 MHz.

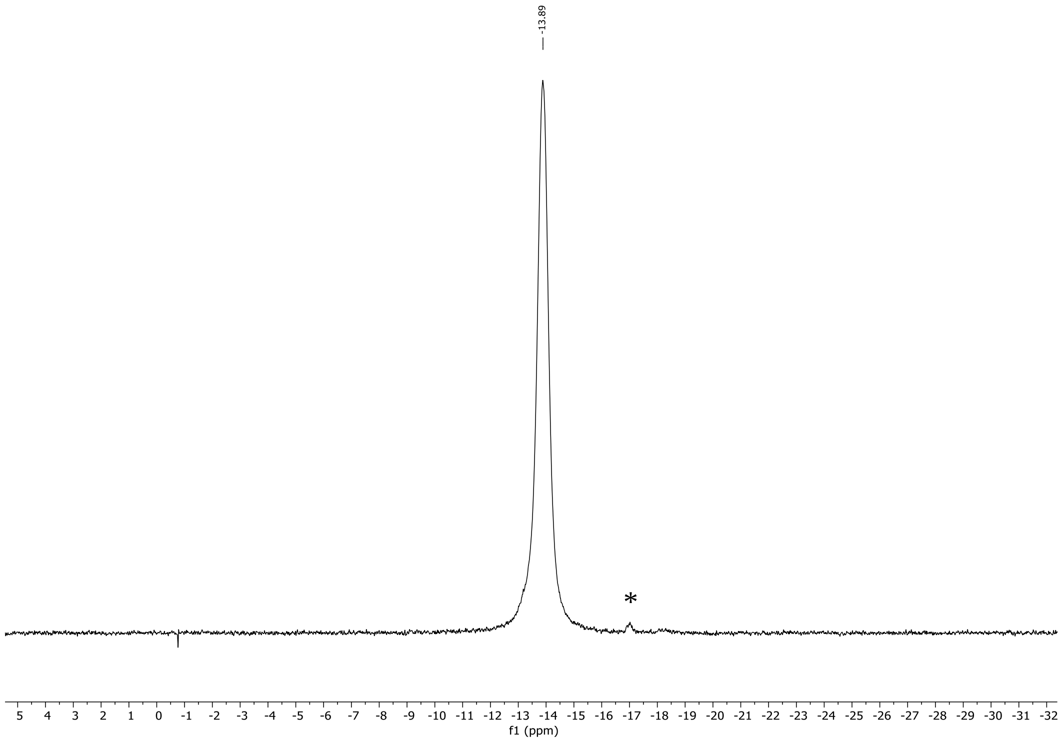

Figure S2. ^{11}B NMR spectrum of $KBEt_3D$ in C_6D_6 at 96 MHz. * marks a small $KBEt_4$ impurity.

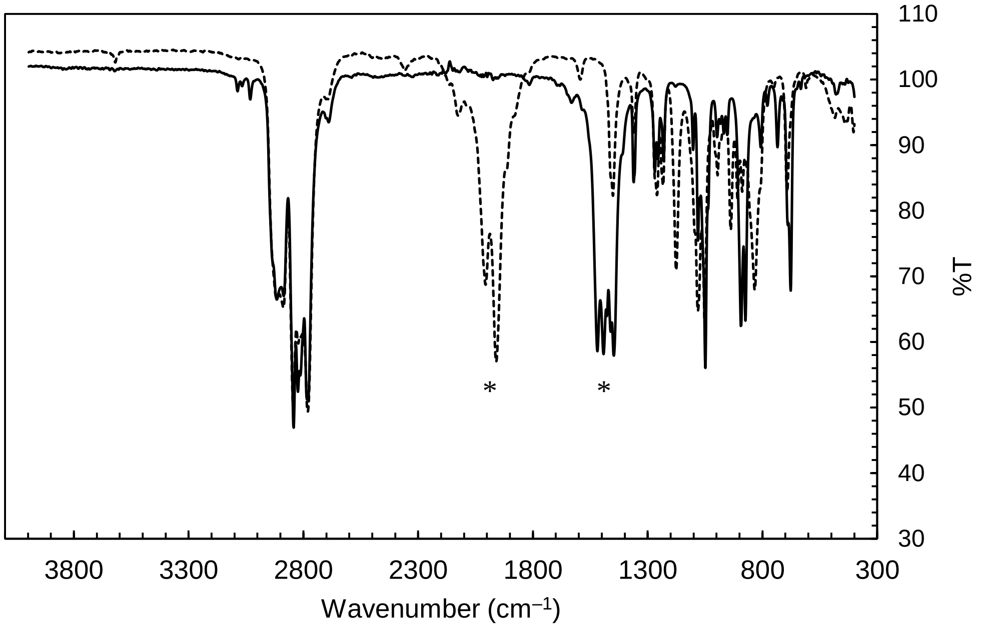

Figure S3. FTIR spectra of $KBEt_3D$ (solid line) and $KBEt_3H$ (dashed line) prepared using the same methodology given in the experimental. * mark B–H/D stretching bands, which are separated by the expect factor of 1.4.

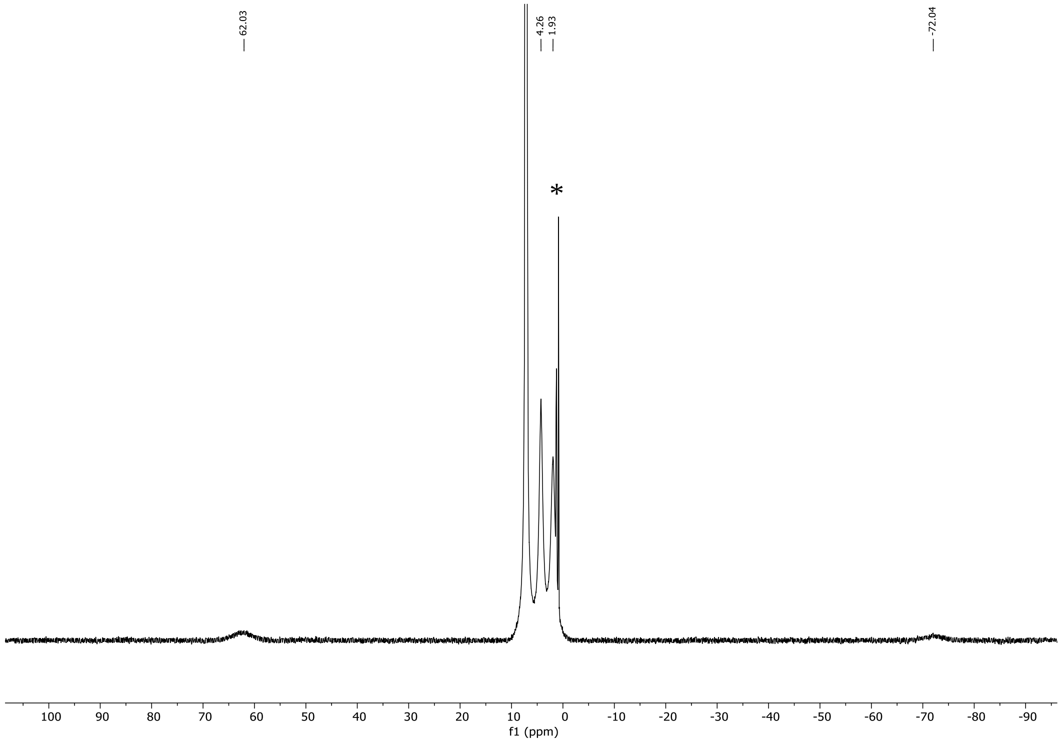

Figure S4. ^{1}H NMR spectrum of (^{tBu}**L**)MnI in C_6D_6 at 400 MHz.

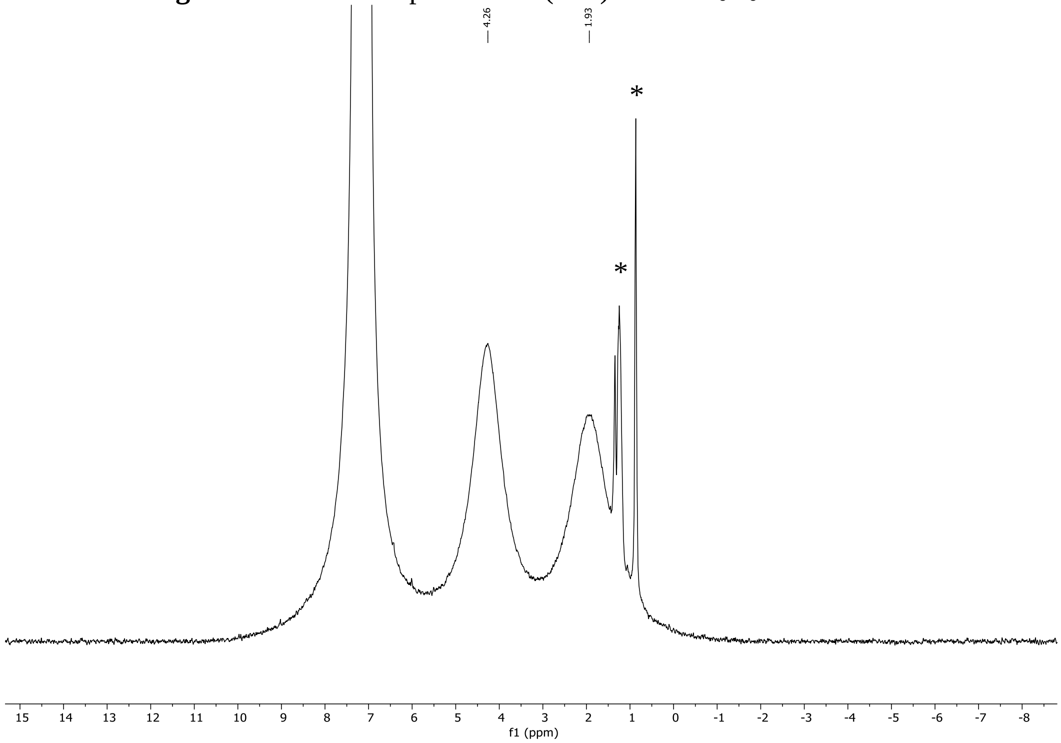

Figure S5. ^{1}H NMR spectrum of (^{tBu}**L**)MnI in C_6D_6 at 400 MHz (close-up of diamagnetic region). * marks a small amount of H-grease that invariably contaminates commercial C_6D_6. This contaminant is particularly apparent here given the relatively low solubility of (^{tBu}**L**)MnI in C_6D_6 and the broadness of the ^{1}H resonances.

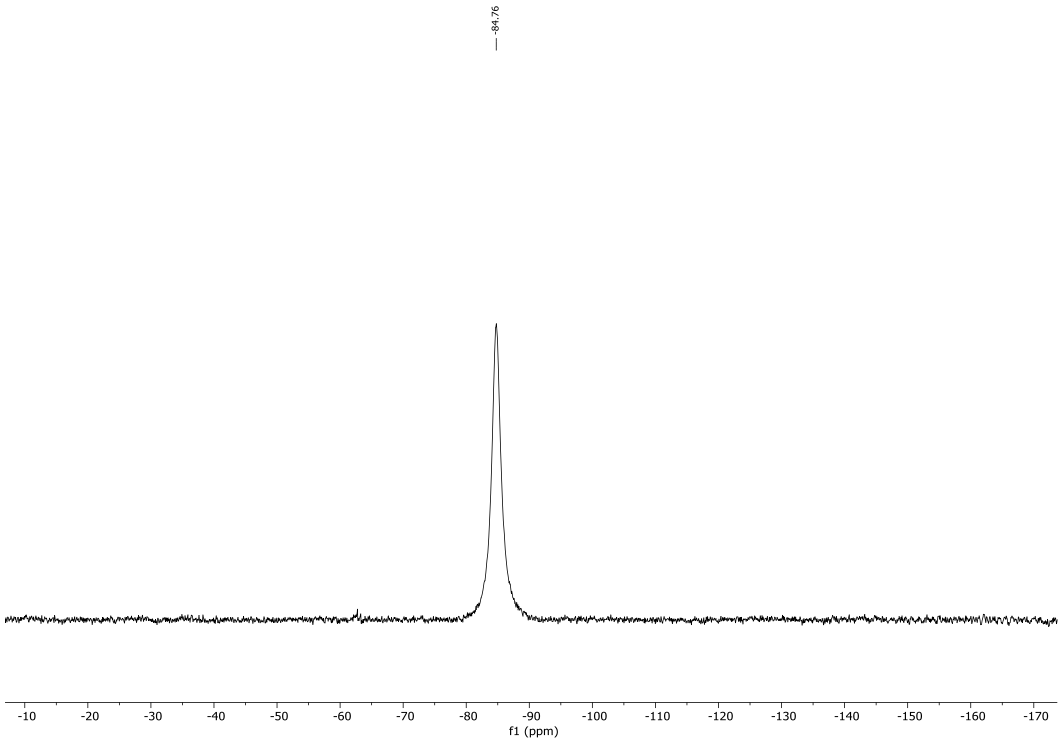

Figure S6. ^{19}F NMR spectrum of (^{tBu}**L**)MnI in C_6D_6 at 376 MHz.

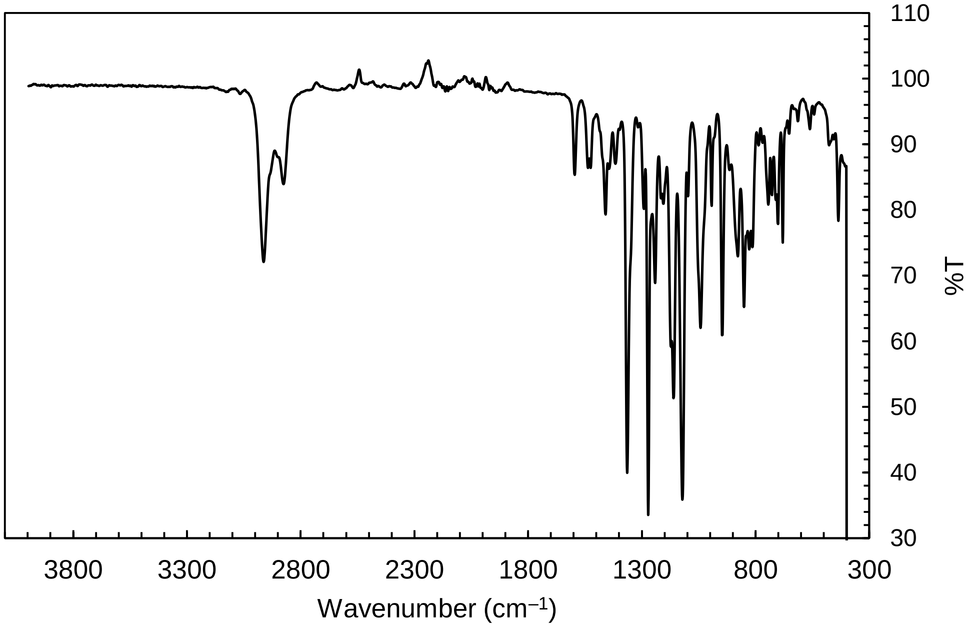

Figure S7. FTIR spectrum of (^{tBu}**L**)MnI.

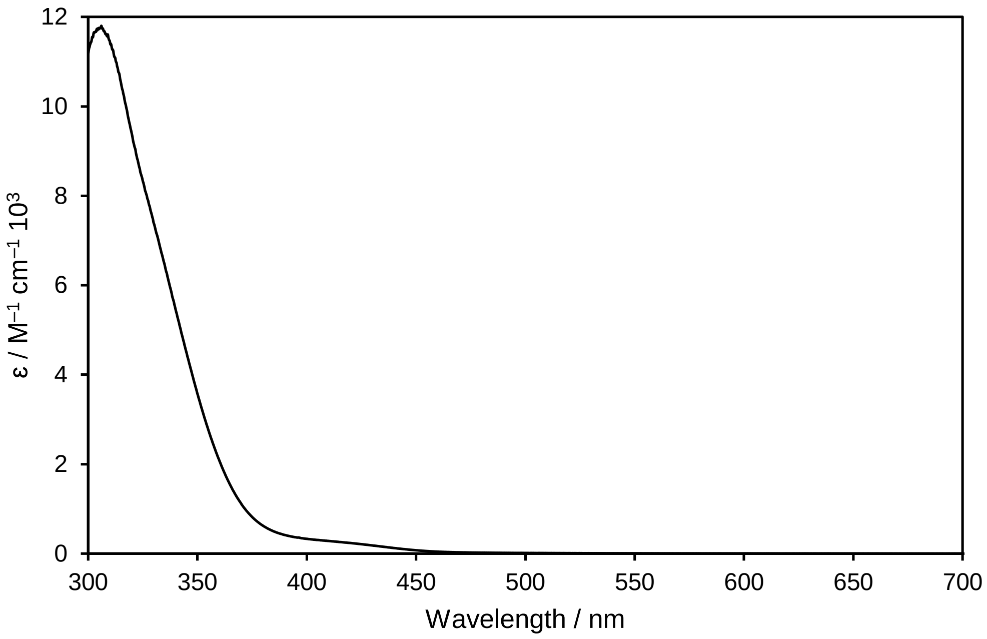

Figure S8. UV-Vis spectrum of (^{tBu}**L**)MnI in C_6H_6.

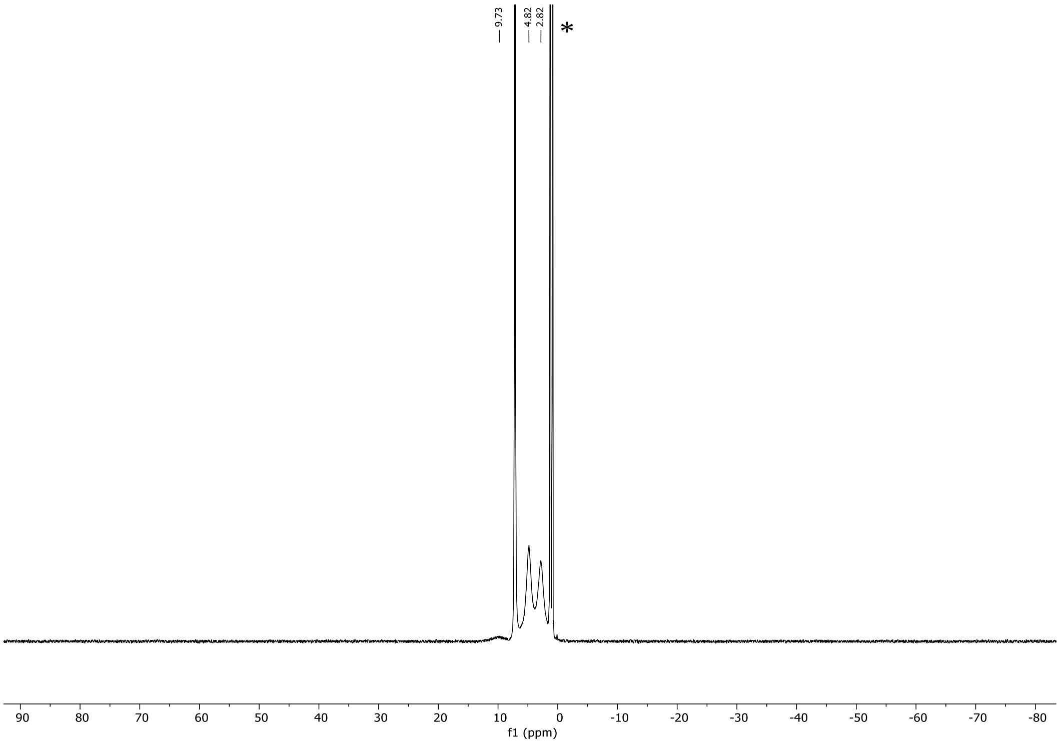

Figure S9. ^1H NMR spectrum of (^{tBu}**L**)MnH in C_6D_6 at 400 MHz. * marks peaks due to *n*-pentane of crystallization.

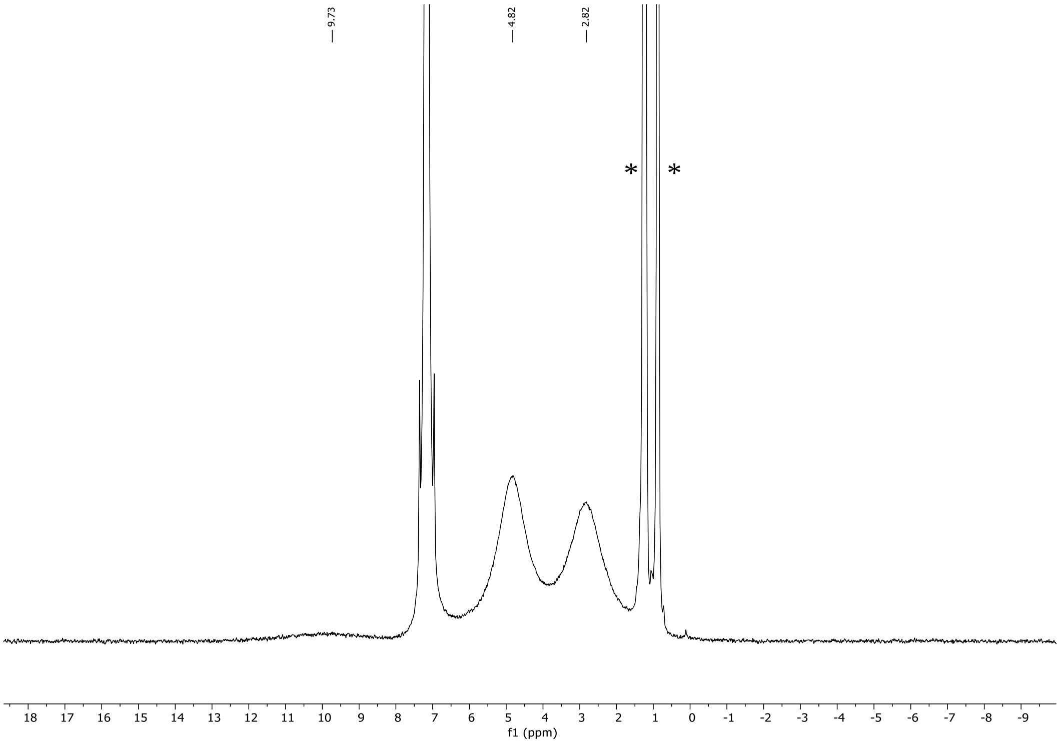

Figure S10. ^1H NMR spectrum of (^{tBu}**L**)MnH in C_6D_6 at 400 MHz (closeup of the diamagnetic region). * marks peaks due to *n*-pentane of crystallization.

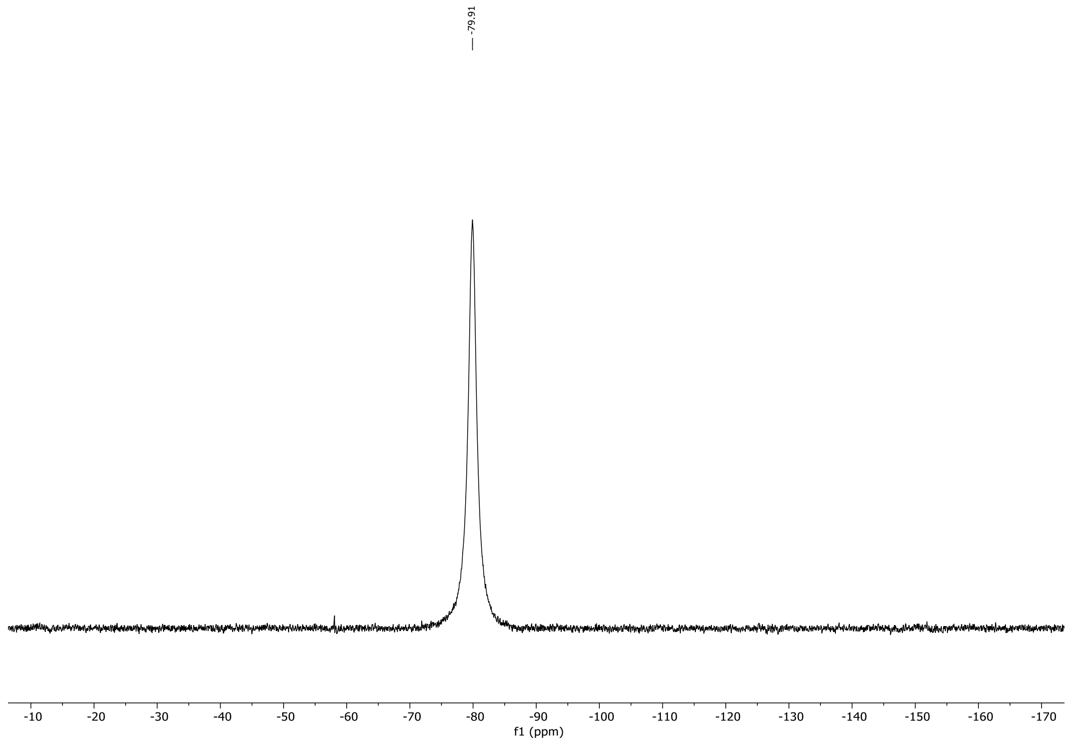

Figure S11. ^{19}F NMR spectrum of (^{tBu}**L**)MnH in C_6D_6 at 376 MHz.

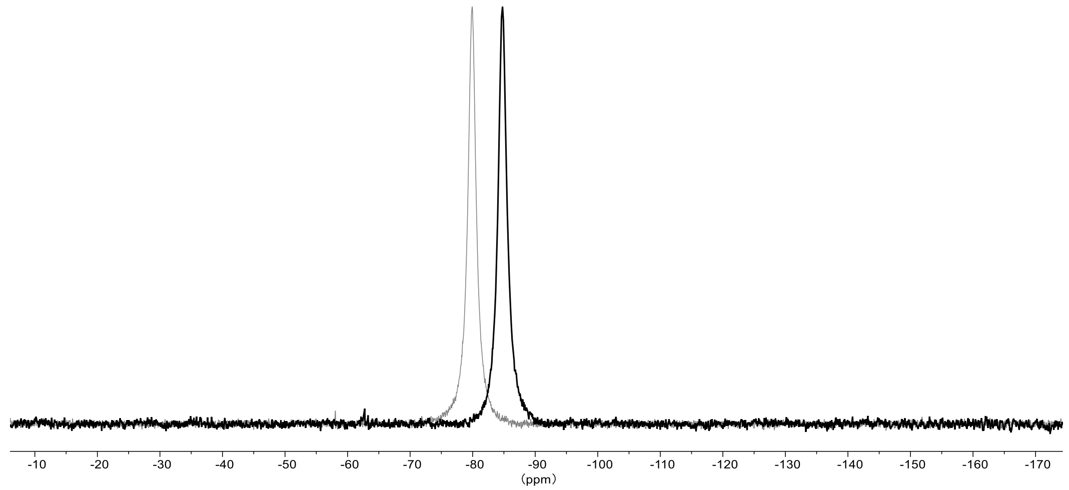

Figure S12. Superimposed ^{19}F NMR spectra of (^{tBu}**L**)MnI (black trace) and (^{tBu}**L**)MnH (grey trace) in C_6D_6 at 376 MHz.

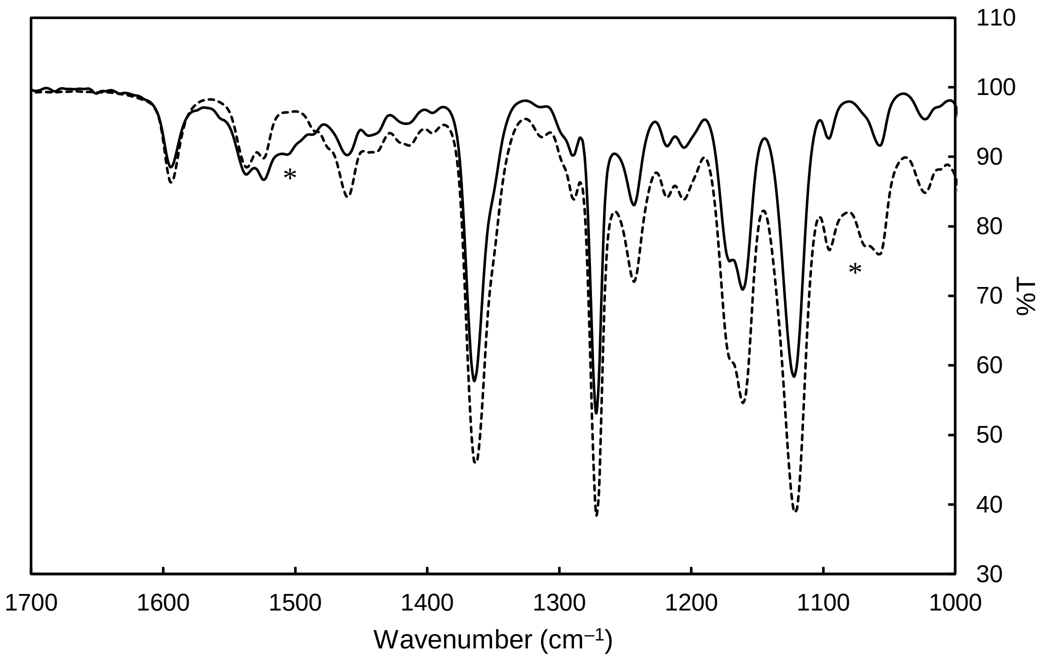

Figure S13. FTIR spectrum of (^{tBu}**L**)MnH (solid line) and (^{tBu}**L**)MnD (dashed line). * mark Mn–H/D stretching bands.

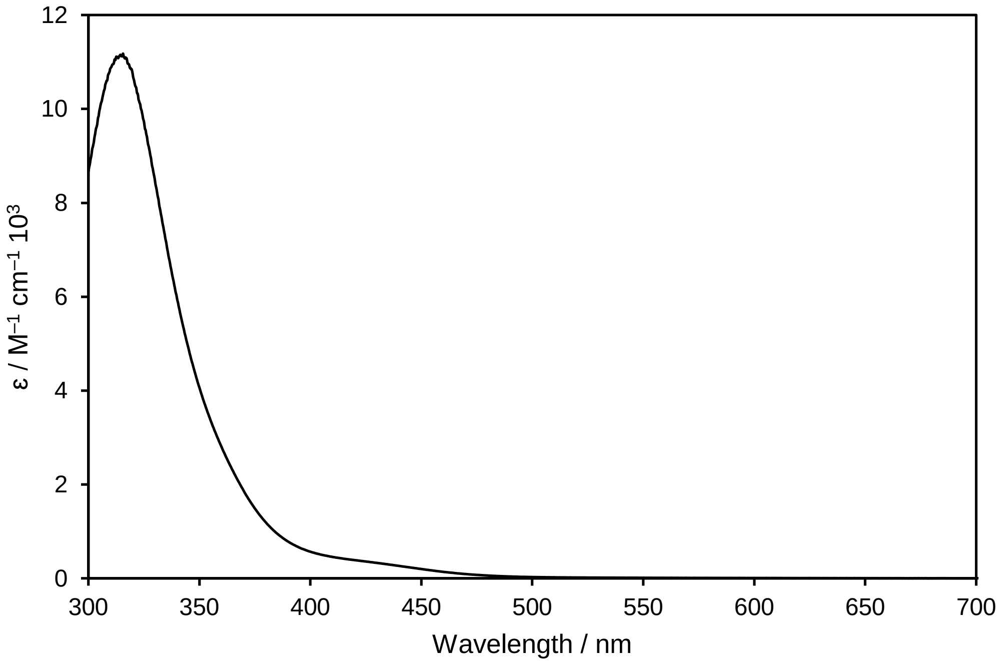

Figure S14. UV-Vis spectrum of (^{tBu}**L**)MnH in C_6H_6.

Additional Data and Discussion

Calculation Details

As shown in Tables S1 and S2, the TPSS family of functionals accurately reproduce the crystallographically-determined coordination geometry about the Mn center for both (^{tBu}**L**)MnI and (^{tBu}**L**)MnH (< 2% error). The two hybrid functionals, TPSSh and TPSS0, with 10% and 25% exact HF exchange, respectively, provide comparable results when considering bond distances and angles; however, TPSS0 clearly provides the most accurate results when considering bond distances alone. This is particularly true for the Mn–C bonds in both complexes, for which both TPSS and TPSSh reliably underestimate the experimentally-observed distances. Therefore, hereafter, all computational results employ the TPSS0-optimized geometries. Notably, both TPSSh and TPSS0 predict the observed elongation of the Mn–C bond upon exchange of iodide for hydride, within 0.01 Å. Subsequent frequency calculations confirm that the geometries optimized at the TPSS0 level are indeed minima.

Table S1. Optimized geometries of (^{tBu}**L**)MnI as a function of %HF.

Parameter	XRD	TPSS (0% HF)	TPSSh (10% HF)	TPSS0 (25% HF)
d(Mn–I) (Å)	2.665(1)	2.624	2.634	2.642
d(Mn–C) (Å)	2.178(2)	2.156	2.165	2.178
d(Mn–N_{pz1} [a]) (Å)	2.184(2)	2.148	2.159	2.168
d(Mn–N_{pz2} [a]) (Å)	2.178(2)	2.157	2.168	2.180
∠(IMnC) (°)	110.86(6)	109.23	109.96	111.11
∠(IMnN_{pz1} [a]) (°)	129.41(5)	130.10	130.10	130.34
∠(IMnN_{pz2} [a]) (°)	126.23(5)	126.95	126.94	127.04
∠(CMnN_{pz1} [a]) (°)	98.94(7)	97.22	97.09	96.01
∠(CMnN_{pz2} [a]) (°)	98.06(7)	101.59	101.62	101.60
∠(N_{pz1}MnN_{pz2}) (°)	86.57(7)	85.42	84.65	83.73
Bond MAPE (%) [b]	–	1.29	0.84	0.42
Total MAPE (%) [b]	–	1.44	1.30	1.31

[a]N_{pz1} is the coordinated pyrazolyl N atom which is proximal to the arene substituent of the alkyl group; N_{pz2} is the distal pyrazolyl N atom. N.B., these are inequivalent due to the presence of the stereogenic alkyl carbon. [b]MAPE = Mean Absolute Percentage Error. For comparison, we present MAPEs determined from bond distances alone (Bond MAPE) as well as including bond angles (Total MAPE).

Table S2. Optimized geometries of (^{tBu}**L**)MnH as a function of %HF.

Parameter	XRD	TPSS (0% HF)	TPSSh (10% HF)	TPSS0 (25% HF)
d(Mn–H) (Å)	1.68(2)	1.68	1.68	1.69
d(Mn–C) (Å)	2.215(1)	2.177	2.193	2.205
d(Mn–N_{pz1} ^{a}) (Å)	2.178(1)	2.147	2.163	2.177
d(Mn–N_{pz2} ^{a}) (Å)	2.176(1)	2.151	2.169	2.183
∠(HMnC) (°)	121.4(8)	121.11	122.76	124.96
∠(HMnN_{pz1} ^{a}) (°)	125.8(8)	125.95	126.00	125.31
∠(HMnN_{pz2} ^{a}) (°)	122.0(8)	120.39	120.14	119.80
∠(CMnN_{pz1} ^{a}) (°)	95.44(5)	95.60	94.93	94.27
∠(CMnN_{pz2} ^{a}) (°)	99.50(5)	100.29	99.59	98.95
∠(N_{pz1}MnN_{pz2}) (°)	84.01(4)	85.41	84.48	83.65
Bond MAPE (%) ^{b}	–	1.07	0.50	0.35
Total MAPE (%) ^{b}	–	0.85	0.60	0.87

^{a}N_{pz1} is the coordinated pyrazolyl N atom which is proximal to the arene substituent of the alkyl group; N_{pz2} is the distal pyrazolyl N atom. N.B., these are inequivalent due to the presence of the stereogenic alkyl carbon. ^{b}MAPE = Mean Absolute Percentage Error. For comparison, we present MAPEs determined from bond distances alone (Bond MAPE) as well as including bond angles (Total MAPE).

Following geometry optimization, selected spectroscopic parameters of (^{tBu}**L**)MnI and (^{tBu}**L**)MnH were computed at a higher level of theory. DFT calculations employed the TPSS0 functional in combination with the enlarged ZORA-def2-TZVPP/ZORA-def2-TZVP(-f) basis set combination (SARC-def2-TZVPP for I), with a fully decontracted SARC/J auxiliary basis. To accurately capture core-polarization effects, the radical integration accuracy was increased (“IntAcc 7”) for all atoms heavier than C, and the C(H) moiety bound to the Mn center, as well as the hydride in the case of (^{tBu}**L**)MnH. Calculation of *g* tensors, ZFS tensors (*D* and *E*/*D*), and hyperfine coupling tensors employed the “eprnmr” module of ORCA, including the effects of spin–spin and spin–orbit interactions. The effects of spin-orbit coupling were treated via a mean-field approach including 1-electron terms, 2-electron Coulomb terms, and the 2-electron exchange term (ORCA flags: “SOCType 3” and “SOCFlags 1, 3, 2, 0”).[27] Tabulated parameters are presented in Table 1 in the main text.

Calculation of Spectroscopic Parameters

For calculation of the *A*-tensor, careful treatment of the core region is required for quantitative prediction of the Fermi contact contribution (i.e., the isotropic component of the tensor). Thus, following the work of Neese,[28-29] we combined dense radial integration grids with recontracted basis sets tailored for

use in a scalar relativistic setting. Given our previous computational work on complexes supported by this ligand system,[1] we expect our DFT method to provide good predictions of the *A*-tensors, which is corroborated in the case of (^{tBu}**L**)MnH for $A(^{1}H)$ (unfortunately, the other *A*-tensors were not resolved experimentally). Given the accurate calculation of the ^{1}H hyperfine coupling tensor for (^{tBu}**L**)MnH, it is interesting to note that $a_{iso}(^{55}Mn)$ for (^{tBu}**L**)MnH is predicted to be roughly two times greater than that of (^{tBu}**L**)MnI, despite the introduction of a ligand, H^{-}, typically assumed to form substantially more covalent bonds with 3d metals than I^{-}. Evidently, this characterization of M–H bonds does not necessarily hold for high-spin ions. Further experiments are planned to better assess the metal–ligand covalencies for (^{tBu}**L**)MnI/H and related species.

Quantitative prediction of *D*-tensors by DFT can be challenging, depending as it does on accurate treatment of the effects of SOC on the ground state. For the specific case of Mn^{2+} (high spin d^5), observed errors in *D* are typically on the order 0.1–0.2 cm^{-1} (3,000–6,000 MHz);[30-31] although, as stated in the main text, heavy element ligand(s) may cause issues with accuracy.[30] To provide a sense of the accuracy of our specific method, we have computed the ZFS parameters for a homologous series of four-coordinate Mn^{2+} complexes $(PPh_3O)_2Mn(X)_2$, where X is a halogen; geometries have been taken from the literature (Table S3).[30]

Table S3. Calculated ZFS parameters for a homologous series of Mn^{2+} complexes.

**Complex**	**|*D*| (cm^{-1})**		***E*/*D***	
	Expt.	DFT	Expt.	DFT
$(PPh_3O)_2Mn(Cl)_2$	0.165	0.170	0.027	0.169
$(PPh_3O)_2Mn(Br)_2$	0.507	0.787	0.264	0.145
$(PPh_3O)_2Mn(I)_2$	0.906	1.768	0.246	0.242

The accuracy of these results is similar to that reported by Neese and co-workers,[30] and demonstrates the decreasing accuracy in |*D*| as the mass of the halogen ligand increases, such that the predicted |*D*| is about a factor of 2 times too large for X = I. Thus, while the systematic trends are captured, which we note in the main text, the quantitative agreement is considerably worse in computing the ZFS for (^{tBu}**L**)MnI.

Finally, we have conducted preliminary calculations on the Fe^{3+} hydride complex isoelectronic to (^{tBu}**L**)MnH; i.e. [(^{tBu}**L**)FeH]$^{+}$. While we note that $|a_{iso}|$ for the two molecules is similar, we defer further discussion of these results until such time as they can be corroborated with experimental data. Experiments to obtain this data are currently being planned.

EPR Spectroscopy

ENDOR Spectroscopy at Low-field edge of Mn-H/D EPR Spectrum. Although we report EasySpin simulation to analyze ENDOR spectra for (^{tBu}**L**)MnH/D in the main text, we present below an alternative approach using perturbation theory to provide insight into the issues that distinguish this system from the many other high-spin Mn^{2+} systems. To begin the development, it is useful to first give a brief introduction to the ^{1,2}H electron-nuclear hyperfine interactions (HFI) that arise in (^{tBu}**L**)MnH/D in the limiting case where the externally applied magnetic field (B) at Q-band makes the electron-Zeeman interaction far larger than the ZFS term, as in for example hexaaquo Mn^{2+}, Mn^{2+} complexes of metabolites, and Mn-lipoxygenase,[23, 32-34] in which case the electron spin quantizes along the direction of B. As the ^{1,2}H HFI is moreover extremely small compared to the electron Zeeman interaction, the HFI can in turn be described by the projection of the hyperfine tensor along the direction of B and is denoted A_B. For the hyperfine coupling of an I = 1/2 (^{1}H) nucleus, or an I = 1 (^{2}H) nucleus without resolved quadrupole splitting, to a high-spin Mn^{2+} (S = 5/2) paramagnetic center that has such weak ZFS interactions, a single molecular orientation ENDOR transition from substate $m_s \rightarrow m_s + 1$ results in a doublet separated by $|A_B|$. The frequencies of the doublet peaks (ν_{ms}, ν_{ms+1}) are given to first order by eqs. S1, where ν_N is the nuclear Larmor frequency. The five electron-spin transitions lead to six possible ENDOR frequencies, but thermal depopulation of higher energy m_s levels at 2 K sharply decreases the number of observed peaks, and indeed we only observe ENDOR responses from the lowest-energy sublevel pair.

$$\nu_{ms} = | \nu_N - m_s A_B |$$

$$\nu_{ms+1} = | \nu_N - m_{s+1} A_B | \qquad \text{(S1a)}$$

$$m_s = \pm 5/2, \pm 3/2, \pm 1/2 \qquad \text{(S1b)}$$

$$\Delta\nu = \nu_{ms} - \nu_{ms+1} = | A_B | \qquad \text{(S1c)}$$

As the ^{2}H spectra do not show resolved quadrupole splittings, these equations likewise describe the frequencies of ^{2}H ($I = 1$) ENDOR transitions.

Eqs. S1 are a result of treating the ZFS interaction as a first-order perturbation on the electron Zeeman interaction, which splits but does not mix the m_s substates. This approach is appropriate when the ZFS is quite small compared to the Zeeman interaction, and m_s therefore can be considered a 'good quantum number'. However, at Q-band microwave frequencies the ZFS in (^{tBu}**L**)MnH/D is neither much less than the Zeeman interaction, where as noted, ENDOR is well-described by eqs. 1, nor is it much larger than the Zeeman interaction as in porphyrins of Mn^{2+} and (more commonly) Fe^{3+}, where the EPR signal is describable as arising from a fictitious spin, $S' = ½$, and the hyperfine interactions are described with a

fictious hyperfine tensor, ***A'***.[35-36] In (^{tBu}**L**)MnH/D, the ZFS terms are sufficiently strong as to require the incorporation of first order mixing of m_s states by the ZFS interaction, and to our knowledge this 'intermediate' behavior has never before been explored. This situation can of course be treated precisely with the general simulation program EasySpin, and ENDOR simulations with this program are presented in the main text. However, doing so does not illuminate the nature and origin of the unusual behavior of the high-spin Mn^{2+} spin centers in (^{tBu}**L**)MnH/D and their ENDOR responses in this parameter regime. Therefore, we present here a perturbation-theory treatment of the single-crystal-like ENDOR responses observed at the low-field edge of the (^{tBu}**L**)MnH/D EPR spectra, which is associated only with the –5/2 → –3/2 manifold (5350 G; Figure 3b). The m_x wavefunctions modified to first order in the ZFS interaction have contributions from m_s + 2 states mixed into the m_s = –5/2, –3/2 states, and as such m_s is no longer a 'good' quantum number. Their use illuminates how the ENDOR results are consequences of this.

The ZFS-induced mixing of m_s sublevels leads to an ENDOR response at the low-field edge of the EPR spectrum that still can be described by equations of precisely the form of eqs. S1, using half-integer constants, –5/2, –3/2, etc., denoted 'm_s'. Instead, the treatment that utilizes the 'm_s' quantum numbers accounts for mixing by incorporating an *effective* hyperfine coupling, A_B', that is *defined* by the observed differences in the peak frequencies of an ENDOR ν_+/ν_- doublet ($\Delta\nu^{obs}$), eq. S2.

$$\Delta\nu^{obs} \equiv | A_B' | \qquad \text{(S2)}$$

How this doublet splitting is altered from the intrinsic coupling associated with the electron-nuclear spin Hamiltonian, A_B, is described in eqs. S4, S5, below.

Interpretation of $A_B' = A_y'$ obtained with field along D_Y. With the external field along the Y-direction of the ZFS tensor (low-field edge of EPR spectrum), treatment of the S = 5/2 Mn^{2+} electron spin with first-order modifications of the m_s = –5/2, –3/2 substates being interrogated by the ZFS interaction yields the wavefunctions for each of the six S = 5/2 spin sublevels, which are denoted as $y_{1\text{-}6}$ in eq. S16 below. In particular, the corrections to the m_s = –5/2 and = –3/2 sublevels arising from the ZFS-mixing give the wavefunctions,

$$|\tfrac{-3}{2}\rangle \rightarrow |y_2\rangle = |\tfrac{-3}{2}\rangle - \sqrt{18}\bullet\Delta|\tfrac{1}{2}\rangle \qquad \text{(S3a)}$$

$$|\tfrac{-5}{2}\rangle \rightarrow |y_1\rangle = |\tfrac{-5}{2}\rangle - \sqrt{10}\bullet\Delta|\tfrac{-1}{2}\rangle \qquad \text{(S3b)}$$

$$\Delta = \frac{\epsilon}{4\beta_e B} \qquad \epsilon = \tfrac{1}{2}(D_{xx} - D_{zz}) \qquad \text{(S3c)}$$

where the parameter Δ incorporates the effect of the axial and rhombic contributions to the ZFS tensor on spectra taken along the ZFS *Y* axis. Electron-spin transitions between these substates in fact give rise to the contribution in the EPR spectrum labelled |–5/2> ↔|–3/2> in Figure 3b. A perturbation-theory computation of the HFI and nuclear Zeeman interaction in these two substates for a nucleus without quadrupole coupling (see below) then yields the ENDOR transition frequencies given in eqs. S4, which incorporate the fact that in the orientation-selective ^{1,2}H ENDOR spectra collected at the low-field edge of the EPR spectrum the hyperfine coupling A_B corresponds to A_y, the hyperfine coupling along the *Y*-axis of the ZFS tensor:

$$|y_2\rangle \qquad \nu_2^{obs} = \left|-\frac{3}{2}A_Y - \nu_N + 9\Delta^2 A_Y\right|$$

$$|y_1\rangle \qquad \nu_1^{obs} = \left|-\frac{5}{2}A_Y - \nu_N - 5\Delta^2 A_Y\right| \qquad (S\mathbf{4})$$

In thus altering the wavefunctions and two ENDOR frequencies, the mixing thereby modifies both the splitting and center-frequency of the ENDOR doublet. As a result, eqs. S5 relates the experimentally observed splitting, A_Y', and the center-frequency of the doublet, ν_{av}, to the intrinsic HFI constant A_Y as modified by the correction factor Δ,

$$\Delta\nu^{obs} = |A_Y(1 + 14\Delta^2)| \equiv |A_Y'| \qquad (S\mathbf{5a})$$

$$\nu_{av} = 2|A_y|(1-\Delta^2) - \nu_N \qquad (S\mathbf{5b})$$

where Δ as calculated with eq. S3c by employing the ZFS parameters used to simulate the EPR spectrum for (^{tBu}**L**)MnH (Figure 2b), *D* = 7600 MHz and *E/D* = 0.15, has the value,

$$\Delta = 0.108 \text{ at } 5{,}350 \text{ G}. \qquad (S\mathbf{5c})$$

Incorporating this value of Δ and the observed ^{2}H doublet splitting for (^{tBu}**L**)MnD, A_Y' = –6 MHz, (Figure 4b) into eqs. S5a,b then yields the spin-Hamiltonian ^{2}H HFI constant, A_Y = –5.2 MHz. In turn, inserting the values for A_Y and Δ into eqs. S4 gives first-order-corrected ENDOR frequencies for the ^{2}H doublet, 3.7 MHz and 9.7 MHz, which agree well with the observed frequencies of 3.5 MHz and 9.5 MHz. This perturbation approach based on ZFS mixing is further supported, and of course readily extended to other fields, by the exact calculations involved in simulation with EasySpin, as described in the main text.

Additional perturbation-treatment details. The spin Hamiltonian operator for an electron-spin whose spin is quantized along the direction of an applied magnetic field (*B*) is given by:

$$\hat{H}_0 = \beta_e g \hat{S}_B B \qquad (S\mathbf{6})$$

where β_e is the Bohr magneton in (Hz T^{-1}) and $\hat{S}_B$ is the effective spin operator oriented along the direction of B. For high-spin Mn^{2+} the g-tensor is treated as isotropic and $g = g_e = 2.00$. Energies for the $S = 5/2$ m_s sublevels are given as:

$$e(m_s) = 2\beta_e B m_s \quad \text{(S7)}$$

In the case of ZFS of intermediate strength compared to the electron Zeeman interaction, the ZFS can be treated as a perturbation whose first-order-corrected energies are given as:

$$\hat{H}_1 = \hat{S}_B \times {}^{B}D \times \hat{S}_B = D_{eff}\, S_B^2 \quad \text{(S8)}$$

$$E_1(m_s) = 2\beta_e B m_s + m_s^2 D_{eff} \quad \text{(S9)}$$

$$D_{eff} = l_1^2 D_1 + l_2^2 D_2 + l_3^2 D_3 \quad \text{(S10)}$$

where $l = (l_1, l_2, l_3)$ are the directional cosines of the applied magnetic field vector in the D-tensor axis system. For treatment of hyperfine couplings, the electron spin states are multiplied by the nuclear states and a further perturbation applied with the electron-nuclear hyperfine interaction (HFI) and isotropic nuclear Zeeman, ignoring possible quadrupole interactions:

$$\hat{H}_2 = S_B \bullet A \bullet I + g_N \beta_N B m_I \quad \text{(S11)}$$

where g_N is the nuclear g-value, β_N is the nuclear magneton, and A is the HFI tensor. When the HFI is small relative to the electron Zeeman, projection of A along the applied field direction gives the following energies for the lower $|-\rangle$ and upper $|+\rangle$ electron spin states m_s and m_{s+1}:

$$\langle + | S_B | + \rangle (m_I A_B) - m_I \nu_N = ((m_s + 1)A_B - \nu_N) m_I$$

$$\langle - | S_B | - \rangle (m_I A_B) - m_I \nu_N = ((m_s) A_B - \nu_N) m_I \quad \text{(S12)}$$

where ν_N is the nuclear Larmor frequency. This is the formalism outlined in eqs. S1 and gives the frequency of the ENDOR transition between the two levels as, $\Delta\nu = A_B$.

When ZFS is significant compared to the electron Zeeman, however, but much less than would require introduction of a fictitious electron spin, the following approach allows for the correction of ENDOR frequencies in the current study. When the magnetic field is applied along the Y-direction of the D-tensor, as in Figure 4, the off-diagonal elements of $\hat{H}_1$ in the $|S, m_s\rangle$ basis set are given as:

$$H_1 = \epsilon(S_1^2 - S_2^2) \quad \text{(S13)}$$

$$\epsilon = ½(D_{xx} – D_{zz}) \quad \text{(S14)}$$

The traceless D-tensor is described in relation to the axial and rhombic ZFS parameters as:

$$D = 3/2(D_{zz})$$

$$E = ½(D_{xx} – D_{yy}) \quad \text{(S15)}$$

First-order ZFS corrections to the $|S, m_s\rangle$ basis set in a magnetic field B gives the first-order- corrected wavefunctions for the six spin substates $y_{1\text{-}6}$ as:

$$|y_6\rangle \text{ » } |\tfrac{5}{2}\rangle - \frac{\sqrt{10}\epsilon}{4\beta_e B}|\tfrac{1}{2}\rangle$$

$$|y_5\rangle \text{ » } |\tfrac{3}{2}\rangle - \frac{\sqrt{10}\epsilon}{4\beta_e B}|\tfrac{-1}{2}\rangle$$

$$|y_4\rangle \text{ » } |\tfrac{1}{2}\rangle - \frac{\sqrt{18}\epsilon}{4\beta_e B}|\tfrac{5}{2}\rangle - \frac{\sqrt{18}\epsilon}{4\beta_e B}|\tfrac{-3}{2}\rangle$$

$$|y_3\rangle \text{ » } |\tfrac{-1}{2}\rangle - \frac{\sqrt{10}\epsilon}{4\beta_e B}|\tfrac{-5}{2}\rangle + \frac{\sqrt{18}\epsilon}{4\beta_e B}|\tfrac{3}{2}\rangle$$

$$|y_2\rangle \text{ » } |\tfrac{-3}{2}\rangle - \frac{\sqrt{18}\epsilon}{4\beta_e B}|\tfrac{1}{2}\rangle$$

$$|y_1\rangle \text{ » } |\tfrac{-5}{2}\rangle - \frac{\sqrt{10}\epsilon}{4\beta_e B}|\tfrac{-1}{2}\rangle \quad \text{(S16)}$$

Applying $\hat{H}_2$ gives the following electron-nuclear energies for the two lowest-energy electron-spin substates y_1 and y_2:

$$\langle y_2| S_B | y_2\rangle(m_I A_Y) – m_I \nu_N = (\tfrac{-3}{2}A_Y - \nu_N + 9\Delta^2 A_Y)m_I$$

$$\langle y_1| S_B | y_1\rangle(m_I A_Y) – m_I \nu_N = (\tfrac{-5}{2}A_Y - \nu_N – 5\Delta^2 A_Y)m_I \quad \text{(S17)}$$

$$\Delta = \frac{\epsilon}{4\beta_e B} \quad \text{(S18)}$$

Where A_Y is the HFI constant projected along the Y-direction of the D-tensor. These in turn give the ENDOR transition frequencies as the absolute $\Delta m_I = \pm 1$ differences in energy for the two lowest-energy electron-spin substates:

$$|y_2\rangle \qquad \nu_2^{obs} = \left|\frac{-3}{2}A_Y - \nu_N + 9\Delta^2 A_Y\right|$$

$$|y_1\rangle \qquad \nu_1^{obs} = \left|\frac{-5}{2}A_Y - \nu_N - 5\Delta^2 A_Y\right| \qquad \text{(S19)}$$

Visualizing the effects of ZFS-induced m_s mixing. The single-crystal-like ^{2}H ENDOR spectrum collected at the low-field edge of the EPR spectrum (Figure S15), displays a hyperfine-split doubled with *apparent* splitting of $A_Y' = \Delta\nu^{obs} = 6.0$ MHz. This doublet is well replicated by EasySpin through use of the electron-spin parameters used to simulate the EPR spectrum (see main text) and the perturbation treatment detailed above, despite the observed splitting being larger than any hyperfine component. If one instead assumed the measured single-crystal-like doublet splitting in Figure S15, $|\Delta\nu^{obs}| = 6$ MHz at 5350 G, equals a true spin-Hamiltonian parameter, A_Y, and that the ZFS is small enough that m_s remains a good quantum number, then according to eqs. S1, ENDOR from the –5/2 → –3/2 manifold would of course give a doublet splitting of –6 MHz, but the center of the doublet would be significantly shifted to higher frequency than observed, as shown in Figure S15.

Determining the sign of A_B. When satellite electron-spin transitions (other than the –1/2 → 1/2) are probed by ENDOR, in particular the –5/2 → –3/2 transition as probed in the single-crystal-like ENDOR spectra of Figure 4, the sign of the effective hyperfine coupling can be obtained using the first-order eqs. S1, despite its limitations in the present case. As seen in Figure S16, for a measured doublet splitting with $|\Delta\nu|/\nu_N \lesssim 2$, as observed for the $^{1/2}$H ENDOR spectra for (^{tBu}**L**)MnH/D in Figure 4, if the coupling were positive the ENDOR doublet would be centered in the vicinity of (4-5)ν_N, counter to what is observed experimentally. The observed centering at ~ 2ν_N instead unambiguously requires the coupling $A_B' < 0$.

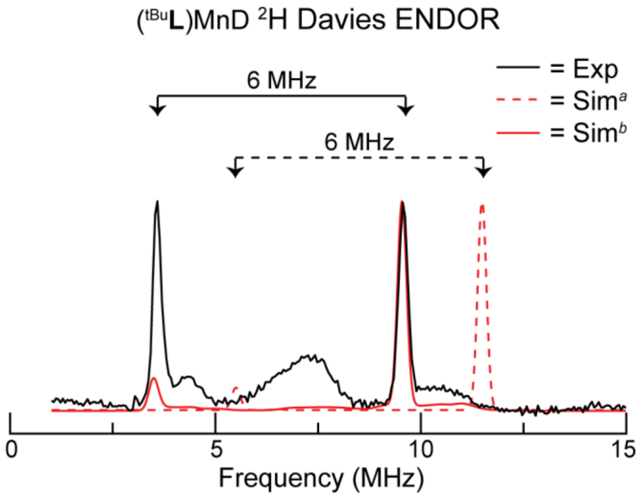

Figure S15. Comparison between the experimentally observed ^{2}H Davies ENDOR (black trace) for (^{tBu}**L**)MnD with simulation using EasySpin (red traces). Experimental spectrum is the same as (^{tBu}**L**)MnD in Figure 3b. Simulation parameters for Sim^a (dashed red trace) are S = 5/2; g = 2; A(^{2}H) = –6 MHz; temperature = 0.5 K; microwave frequency = 35 GHz; magnetic field = 5350 G; ENDOR linewidth = 0.25 MHz; excitation width = 300 MHz; Hstrain = 250 MHz. Parameters for Sim^b (solid red trace) are the same as for Sim^a but with D = [–1363, –3703, 5067] MHz; A(^{55}Mn) = -250 MHz; A(^{2}H) = [–5.32, –5.32, 1.0] MHz, and temperature = 2 K.

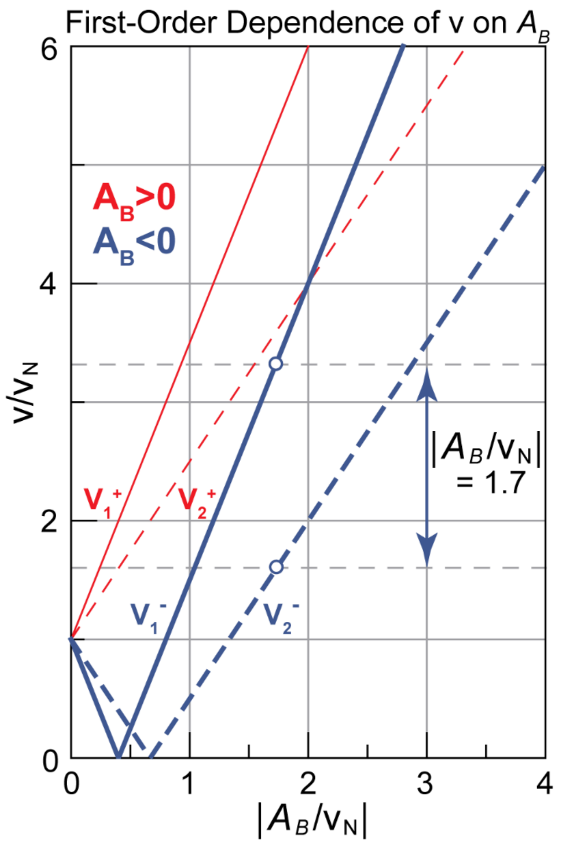

Figure S16. Dependence of ENDOR frequencies (ν) on A_B for m_s = –5/2 (ν_1, solid lines) and m_s = –3/2 (ν_2, dashed lines) levels using the first-order eqs S1. ν_N is the nuclear Larmor frequency. Red lines correspond to ENDOR frequencies if A_B is positive ($\nu_{1,2}^+$), and blue lines correspond to ENDOR frequencies if A_B is negative ($\nu_{1,2}^-$). Blue circles and arrow correspond to ENDOR frequencies and splitting respectively for $|A_B|$ = 6 MHz and ν_N = 3.5 MHz if A_B is negative. This gives ENDOR frequencies at 5.5 MHz and 11.5 MHz, after multiplying by ν_N.

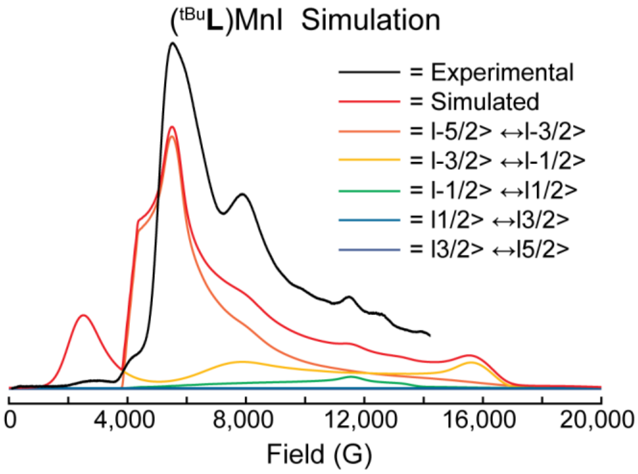

Figure S17. 2 K 35 GHz CW absorption-display EPR spectrum of (^{tBu}**L**)MnI (black trace) with simulation (red trace) and the contributions from individual transitions differentiated by color. Parameters used for simulation are *D*: 24,000 MHz (0.83 cm^{-1}); *E/D*: 0.03; *A*: 250 MHz; *f*: 0.1.

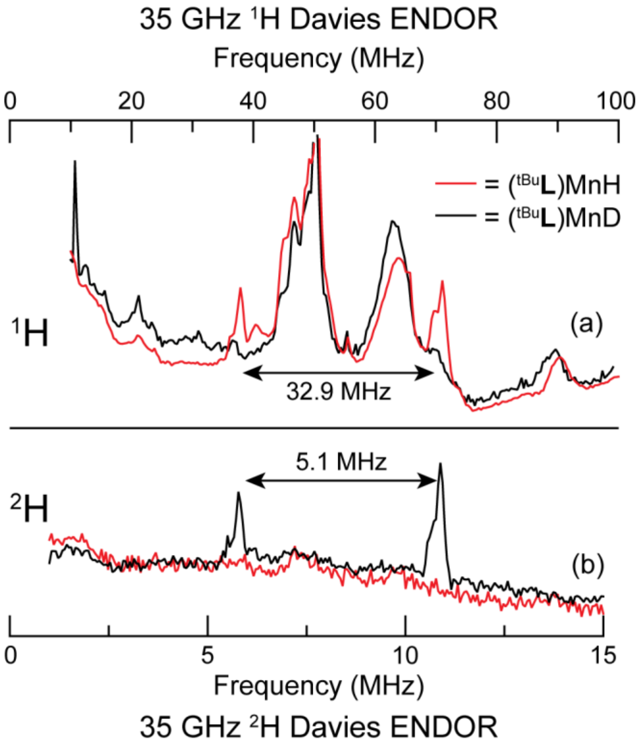

Figure S18. (a) 35 GHz ^{1}H Davies ENDOR spectra at 11.8 kG and 2K of (^{tBu}**L**)MnH (red trace) and (^{tBu}**L**)MnD (black trace). (b) 35 GHz ^{2}H Davies ENDOR at 11.8 kG of (^{tBu}**L**)MnH (red trace) and (^{tBu}**L**)MnD (black trace). Frequency axis in (b) is scaled by a factor of 6.5 to account for the difference in g_n between ^{1}H and ^{2}H. Experimental conditions as in Figure 3. Spectra normalized for clarity.

References

(1) McSkimming, A.; Thompson, N. B., Four-Coordinate Fe N_2 and Imido Complexes Supported by a Hemilabile NNC Heteroscorpionate Ligand. *Inorg. Chem.* **2022**, *61* (31), 12318-12326.

(2) Rosenau, C. P.; Jelier, B. J.; Gossert, A. D.; Togni, A., Exposing the Origins of Irreproducibility in Fluorine NMR Spectroscopy. *Angew. Chem. Int. Ed.* **2018**, *57* (30), 9528-9533.

(3) Schubert, E. M., Utilizing the Evans Method with a Superconducting NMR Spectrometer in the Undergraduate Laboratory. *J. Chem. Educ.* **1992**, *69* (1), 62-62.

(4) Bain, G. A.; Berry, J. F., Diamagnetic Corrections and Pascal's Constants. *J. Chem. Educ.* **2008**, *85* (4), 532-536.

(5) SADABS, v2014/5; Bruker AXS Inc.: Madison, WI, **2001**.

(6) Krause, L.; Herbst-Irmer, R.; Sheldrick, G. M.; Stalke, D., Comparison of Silver and Molybdenum Microfocus X-Ray Sources for Single-Crystal Structure Determination. *J. Appl. Cryst.* **2015**, *48*, 3-10.

(7) Sheldrick, G. M., ShelXT - Integrated Space-Group and Crystal-Structure Determination. *Acta Cryst. A* **2015**, *71*, 3-8.

(8) Sheldrick, G. M., Crystal Structure Refinement with ShelXL. *Acta Cryst. C* **2015**, *71*, 3-8.

(9) Muller, P., Practical Suggestions for Better Crystal Structures. *Cryst. Rev.* **2009**, *15* (1), 57-83.

(10) Neese, F., The ORCA Program System. *Wires Comp. Mol. Sci.* **2012**, *2* (1), 73-78.

(11) Tao, J. M.; Perdew, J. P.; Staroverov, V. N.; Scuseria, G. E., Climbing the Density Functional Ladder: Nonempirical Meta-Generalized Gradient Approximation Designed for Molecules and Solids. *Phys. Rev. Lett.* **2003**, *91* (14).

(12) Staroverov, V. N.; Scuseria, G. E.; Tao, J. M.; Perdew, J. P., Comparative Assessment of a New Nonempirical Density Functional: Molecules and Hydrogen-Bonded Complexes. *J. Chem. Phys.* **2003**, *119* (23), 12129-12137.

(13) Grimme, S., Accurate Calculation of the Heats of Formation for Large Main Group Compounds with Spin-Component Scaled MP2 Methods. *J. Phys. Chem. A* **2005**, *109* (13), 3067-3077.

(14) vanLenthe, E.; vanLeeuwen, R.; Baerends, E. J.; Snijders, J. G., Relativistic Regular Two-Component Hamiltonians. *Int. J. Quantum Chem.* **1996**, *57* (3), 281-293.

(15) Grimme, S.; Antony, J.; Ehrlich, S.; Krieg, H., A Consistent and Accurate Ab Initio Parametrization of Density Functional Dispersion Correction (DFT-D) for the 94 Elements H-Pu. *J. Chem. Phys.* **2010**, *132* (15), 154104-1-19.

(16) Grimme, S.; Ehrlich, S.; Goerigk, L., Effect of the Damping Function in Dispersion Corrected Density Functional Theory. *J. Comp. Chem.* **2011**, *32* (7), 1456-1465.

(17) Pantazis, D. A.; Chen, X. Y.; Landis, C. R.; Neese, F., All-Electron Scalar Relativistic Basis Sets for Third-Row Transition Metal Atoms. *J. Chem. Theory Comp.* **2008**, *4* (6), 908-919.

(18) Rolfes, J. D.; Neese, F.; Pantazis, D. A., All-Electron Scalar Relativistic Basis Sets for the Elements Rb-Xe. *J. Comp. Chem.* **2020**, *41* (20), 1842-1849.

(19) Weigend, F., Accurate Coulomb-Fitting Basis Sets for H to Rn. *Phys. Chem. Chem. Phys.* **2006**, *8* (9), 1057-1065.

(20) Neese, F.; Wennmohs, F.; Hansen, A.; Becker, U., Efficient, Approximate and Parallel Hartree-Fock and Hybrid DFT Calculations. A 'Chain-of-Spheres' Algorithm for the Hartree-Fock Exchange. *Chem. Phys.* **2009**, *356* (1-3), 98-109.

(21) Werst, M. M.; Davoust, C. E.; Hoffman, B. M., Ligand Spin-Densities in Blue Copper Proteins by Q-Band ^{1}H and ^{14}N ENDOR Spectroscopy. *J. Am. Chem. Soc.* **1991**, *113* (5), 1533-1538.

(22) Stoll, S.; Schweiger, A., Easyspin, a Comprehensive Software Package for Spectral Simulation and Analysis in EPR. *J. Mag. Reson.* **2006**, *178* (1), 42-55.

(23) Horitani, M.; Offenbacher, A. R.; Carr, C. A. M.; Yu, T.; Hoeke, V.; Cutsail, G. E.; Hammes-Schiffer, S.; Klinman, J. P.; Hoffman, B. M., C ENDOR Spectroscopy of Lipoxygenase-Substrate Complexes Reveals the Structural Basis for C-H Activation by Tunneling. *J. Am. Chem. Soc.* **2017**, *139* (5), 1984-1997.

(24) Davoust, C. E.; Doan, P. E.; Hoffman, B. M., Q-Band Pulsed Electron Spin-Echo Spectrometer and Its Application to ENDOR and ESEEM. *J. Magn. Reson. Ser. A* **1996**, *119* (1), 38-44.

(25) Epel, B.; Gromov, I.; Stoll, S.; Schweiger, A.; Goldfarb, D., Spectrometer Manager: A Versatile Control Software for Pulse EPR Spectrometers. *Concept Mag. Reson. B* **2005**, *26B* (1), 36-45.

(26) Fryzuk, M. D.; Lloyd, B. R.; Clentsmith, G. K. B.; Rettig, S. J., Binuclear Palladium Complexes with Bridging Hydrides - Unusual Coordination Behavior of $LiBEt_4$ and $NaBEt_4$. *J. Am. Chem. Soc.* **1994**, *116* (9), 3804-3812.

(27) Neese, F., Efficient and Accurate Approximations to the Molecular Spin-Orbit Coupling Operator and Their Use in Molecular-Tensor Calculations *J. Chem. Phys.* **2005**, *122* (3).

(28) Neese, F., Prediction and Interpretation of the Fe Isomer Shift in Mossbauer Spectra by Density Functional Theory. *Inorg. Chim. Acta* **2002**, *337*, 181-192.

(29) Neese, F., Metal and Ligand Hyperfine Couplings in Transition Metal Complexes: The Effect of Spin-Orbit Coupling as Studied by Coupled Perturbed Kohn-Sham Theory. *J. Chem. Phys.* **2003**, *118* (9), 3939-3948.

(30) Zein, S.; Duboc, C.; Lubitz, W.; Neese, F., A Systematic Density Functional Study of the Zero-Field Splitting in Mn(II) Coordination Compounds. *Inorg. Chem.* **2008**, *47* (1), 134-142.

(31) Zein, S.; Neese, F., Ab Initio and Coupled-Perturbed Density Functional Theory Estimation of Zero-Field Splittings in Mn Transition Metal Complexes. *J. Phys. Chem. A* **2008**, *112* (34), 7976-7983.

(32) Tan, X. L.; Bernardo, M.; Thomann, H.; Scholes, C. P., Pulsed and Continuous Wave Electron Nuclear Double-Resonance Patterns of Aquo Protons Coordinated in Frozen Solution to High-Spin Mn^{2+}. *J. Chem. Phys.* **1993**, *98* (7), 5147-5157.
(33) Manikandan, P.; Carmieli, R.; Shane, T.; Kalb, A. J.; Goldfarb, D., W-Band ENDOR Investigation of the Manganese-Binding Site of Concanavalin A: Determination of Proton Hyperfine Couplings and Their Signs. *J. Am. Chem. Soc.* **2000**, *122* (14), 3488-3494.
(34) McNaughton, R. L.; Reddi, A. R.; Clement, M. H. S.; Sharma, A.; Barnese, K.; Rosenfeld, L.; Gralla, E. B.; Valentine, J. S.; Culotta, V. C.; Hoffman, B. M., Probing in Vivo Mn Speciation and Oxidative Stress Resistance in Yeast Cells with Electron-Nuclear Double Resonance Spectroscopy. *Proc. Nat. Acad. Sci. USA* **2010**, *107* (35), 15335-15339.
(35) Hoffman, B. M.; Weschler, C. J.; Basolo, F., Dioxygen Adduct of Meso-Tetraphenylporphyrin manganese(II), a Synthetic Oxygen Carrier. *J. Am. Chem. Soc.* **1976**, *98* (18), 5473-5482.
(36) Scholes, C. P.; Lapidot, A.; Mascarenhas, R.; Inubushi, T.; Isaacson, R. A.; Feher, G., Electron Nuclear Double-Resonance (ENDOR) from Heme and Histidine Nitrogens in Single-Crystals of Aquometmyoglobin. *J. Am. Chem. Soc.* **1982**, *104* (10), 2724-2735.
